# De novo assembly and comparative genome analysis for polyhydroxyalkanoates-producing *Bacillus* sp. BNPI-92 strain

**DOI:** 10.1186/s43141-023-00578-7

**Published:** 2023-11-22

**Authors:** Seid Mohammed Ebu, Lopamudra Ray, Ananta N. Panda, Sudhansu K. Gouda

**Affiliations:** 1 Department of Applied Biology, SoANS, Adama Science and Technology University, Oromia, Ethiopia; 2 School of Law, Campus -16 Adjunct Faculty, School of Biotech, Campus-11 KIIT University, Bhubaneswar, Odisha 751024 India; 3 School of Biotechnology, Campus-11 KIIT University, Bhubaneswar, Odisha 751024 India

**Keywords:** ANI, *Bacillus* species, Genome DNA, Gene, Metabolic pathway

## Abstract

**Background:**

Certain *Bacillus* species play a vital role in polyhydroxyalkanoate (PHA) production. However, most of these isolates did not properly identify to species level when scientifically had been reported.

**Results:**

From NGS analysis, 5719 genes were predicted in the de novo genome assembly. Based on genome annotation using RAST server, 5,527,513 bp sequences were predicted with 5679 bp number of protein-coding sequence. Its genome sequence contains 35.1% and 156 GC content and contigs, respectively. In RAST server analysis, subsystem (43%) and non-subsystem coverage (57%) were generated. Ortho Venn comparative genome analysis indicated that *Bacillus* sp. BNPI-92 shared 2930 gene cluster (core gene) with *B. cereus* ATCC 14579^ T^ (AE016877), *B. paranthracis* Mn5T (MACE01000012), *B. thuringiensis* ATCC 10792 T (ACNF01000156), and *B. antrics* Amen T (AE016879) strains. For our strain, the maximum gene cluster (190) was shared with *B. cereus* ATCC 14579^ T^ (AE016877). For Ortho Venn pair wise analysis, the maximum overlapping gene clusters thresholds have been detected between *Bacillus* s p.BNPI-92 and *Ba. cereus* ATCC 14579^ T^ (5414). Average nucleotide identity (ANI) such as OriginalANI and OrthoANI, in silicon digital DND-DNA hybridization (*is*DDH), Type (Strain) Genome Server (TYGS), and Genome-Genome Distance Calculator (GGDC) were more essentially related *Bacillus* sp. BNPI-92 with *B. cereus* ATCC 14579^ T^ strain. Therefore, based on the combination of RAST annotation, OrthoVenn server, ANI and isDDH result *Bacillus* sp.BNPI-92 strain was strongly confirmed to be a *B. cereus* type strain. It was designated as *B. cereus* BNPI-92 strain. In *B. cereus* BNPI-92 strain whole genome sequence, PHA biosynthesis encoding genes such as *phaP*, *phaQ, phaR* (PHA synthesis repressor *phaR* gene sequence), *phaB/phbB*, and *phaC* were predicted on the same operon. These gene clusters were designated as *phaPQRBC*. However, *phaA* was located on other operons.

**Conclusions:**

This newly obtained isolate was found to be new a strain based on comparative genomic analysis and it was also observed as a potential candidate for PHA biosynthesis.

**Supplementary Information:**

The online version contains supplementary material available at 10.1186/s43141-023-00578-7.

## Background

*Bacillus* is one of the most diverse and versatile genus having representative members that has been reported from different natural ecosystems. A number of *Bacillus* species have been a well-known PHA-producing bacterial cells [1]. For instance, a newly isolated polyhydroxyalkanoates (PHA)-producing *Bacillus* sp. was identified to be a strain of *B. cereus* using microbiological and molecular techniques. It was designated as *B. cereus* SPV [2]. Gram-positive *Bacillus* has rarely been reported for polyhydroxyalkanoates (PHAs) at industrial levels in spite of the fact that a number of Gram-negative bacteria have been known for industrial applications [3]. There is similarly another newly characterized *B. cereus* strain tsu1 that has been identified by draft genome sequencing for PHA biosynthesis. Further study confirmed that *B. cereus* SE-1 and *Bacillus* sp*.* CS-605 were able to accumulate PHA [4]. It has been observed that PHA accumulation was increased for isolate *B. cereus* SE-1 than *Bacillus* sp. CS-605 under the same condition after 72 h of incubation.

*B. cereus* and *B. megaterium* have also been well-known as PHA accumulators [5]. These accumulated PHA which can be biologically degraded have been used for various applications [6]. It was also confirmed that *Bacillus* sp. LPPI-18 which gram-positive isolates has been employed for PHA production [7]. They harbor genomic attributes (genes encoding proteins, responsible) for bioplastic production. Literature surveys revealed differences in the details of these genomic attributes for PHA metabolism. For instance, genes encoding MaoC-like protein have been reported in the *B. cereus* that contains the *pha* gene cluster. However, this protein was not detected in *B. megaterium*. Literature review revealed that this protein has been used to encode Enoyl-CoA hydratase (R-hydratase) enzymes that are involved in PHA metabolism [5]. The poly (3-hydroxybutyrate-co-3-hydroxyvalerate) (PHA) biosynthesis can also be improved after genetic engineering from certain carbon sources such as glucose and propionic acid using *E.coli* [8]*.* Comparative genomic analysis has been used for study of relationships among different homologous genes such as orthologs gene that originate from a common ancestor during speciation events and paralogs that also share a common ancestor but arise from sequence duplication events within a species. Orthologous genes are usually syntenic between close-related species. Whereas, paralogs synteny genes often show a limited and more speciation-related divergence. Comparative genomic study used to compare multiple species with high sequence similarity due to orthologous genes that function with comparable biological function. It is also used to compare the genome of species that are able to perform distinct functions due to sequence with greater divergence from other species. On the other hand, orthologs with sequences that show are more likely to perform distinct functions [9–11].

Genomic DNA analysis is also a major source of information for microbial identification in microbial taxonomy [12]. For instance, certain pathogenic bacterial species like *Bordetella petrii* [13] can be differentiated based on genomic DNA analysis*.* It has been used for high-throughput DNA sequencing technologies [12]. Nowadays, the major application of genomic DNA sequence is to measure overall genomic relatedness between two microbial strains. It has also served as the framework for the species concept [14]. For microbial identification, the DNA–DNA hybridization (DDH) method is well-known for certain years and considered as a gold standard for microbial taxonomy [15] in spite of indirectly measuring genome sequence similarity, labor-intensive, and error-prone [16]. However, currently, several overall genome relatedness indices have been developed for microbial identification using whole-genome sequencing that has been freely available. Hence, OGRI used to replace costly DDH methods and is able to calculate the similarity between two genomic DNA sequences without gene-finding and functional reproducible, fast, and easy-to-implement [17].

Orthologous Average Nucleotide Identity (OAT) is another comparative genome analysis tool that is able to use OrthoANI to measure similarity between two genomic sequences. ANI and OrthoANI are comparable algorithms. They share the same species demarcation cut-off at 95 ~ 96% and large comparison studies have demonstrated both algorithms to produce near identical reciprocal similarities. OrthoANI is highly correlated with ANI (using BLASTn) and the former showed approximately 0.1% higher values than the latter. OrthoANI is more commonly used for calculating ANI similarity between two or more microbial strains. It is more robust and faster for taxonomic determinations. The standalone software tools are freely available at http://www.ezbiocloud.net/sw/oat. In fact, OAT employs an easy-to-follow Graphical User Interface that allows researchers to calculate OrthoANI values between genomes of interest [17]. They share the same species demarcation cut-off at 95 ~ 96% and large comparison studies have demonstrated both algorithms to produce near identical reciprocal similarities. OrthoANI is highly correlated with ANI (using BLASTn) and the former showed approximately 0.1% higher values than the latter.

Comparative genome analysis is also performed by certain software like OrthoVenn, which is one of the web-based platforms used for comparison and annotation of orthologous gene clusters among multiple microbial species. It works on any operating system with a modern browser and Javascript-enabled. OrthoVenn is used to provide a comprehensive coverage of bacteria, fungi, metazoan, protists, plants, and vertebrates for identification of orthologous gene clusters and supports users for uploading protein sequences. OrthoVenn has an efficient and interactive graphic tool which provides a Venn diagram view for comparing protein sequences that belong to multiple microbial species [18]. The only things users need to do are choosing species or upload protein sequences.

The draft genome sequence (5.81 Mb) and protein-coding sequence (5673 f) have also been used to predict genomic information of certain microbes and hence employed for microbial identification. Genes involved in cellulose degradation and PHA biosynthesis pathways have likely been reported for this strain. Similarly, 8 rRNA genes such as 5S, 16S, and 23S have been detected in this genome draft [19].

Certain PHA-producing microbial cells with *pha* genes have been reported and identified by using de novo assembly which is a fundamental technique for identification of various microbial diversity used for different fields of applications. For instance, de novo assembly is a foundation for the development of genetic resources such as gene prediction, high-resolution maps of polymorphism, and genomic structural variation [20]. In addition to *pha* genes, certain other genetic information have been predicted for this isolate with different features.

These genomic features have been achieved with genomic DNA annotation, a process of extraction of biological information from a series of nucleotide sequencing data and identification of their respective role in biological systems which is a similar work with [21]. So far, two main levels of genome annotation have been identified [22]. These two-component hierarchies of genome annotations have been included such as a static view of genome annotation and a dynamic view of genome annotation [23].

Very few PHA-producing de novo genome assembly for *Bacillus* strains have been reported till date. Therefore, to fulfill this gap of knowledge, the present study was aimed to perform de novo assembly and comparative genomic analyses for PHA-producing *Bacillus* sp. BNPI-92 strain obtained from an area of plastic waste accumulation. It is also to identify location of evolutionary relationships, the genetic basis associated, and level of gene expression for this PHA-producing strain using different software. The other gap of knowledge to be fulfilled in this study is to identify orthologous genes and determine the degree of sequence similarity among or between microbial cells and identification of gene ontology (GO) involved in encoding gene clusters used for biosynthesis of PHA granules. The identification of protein-encoding for PHA metabolisms and its location and identification of BNPI-92 strain based on ANI and GGDC 2.1 using genome–genome distance phylogeny (GGDP) methods are also few important methods of comparative genomics studies to understand the evolutionary history of this strain and calculate their intergenomic distance.

## Methods

Whole-genome sequence (WGS) was performed using next-generation sequencer (NGS). Briefly, Fastq quality checking and filtering, de *novo* genome assembly, gene prediction, and gene annotation were performed. Quality checking such as base quality score distribution, average base content per read, and GC distribution in the reads were performed for an input fastq file. The fastq files were pre-processed before performing the assembly. The adapter sequences were trimmed using the Trimmomatic tool. It then filtered out reads with an average quality score less than 30 in any of the paired-end reads [18, 24].

## Genome assembly and genome coverage

De novo assembly was performed using SPAdes, ABySS, and Velvet software. The default k-mer sizes were used for SPAdes assembly. A range of k-mers from 31 to 95 was used for Velvet assembly and ABySS assembly. Velvet assembler was used with k-mer 47 for all further downstream analysis since it has better statistics than all other assemblies generated using QUAST 4.0 and BUSCO 2.0 statistical tools [25–29]. The genome coverage for the recent our isolate was also calculated using genome coverage = (read count * read length) / total genome size.

## Gene prediction and gene annotation

The genes were predicted from the Velvet assembled contigs using Glimmer software. It was available at https://ccb.jhu.edu/software/glimmer/. The predicted genes were annotated using in-house pipeline RAST server that is available at http://rast.theseed.org/FIG/rast.cgi.

### Matching with UniProt database using BLASTX program

The predicted genes were compared with UniProt database using BLASTX program with E-value cutoff of 10^–3^. It was available at ftp://ftp.ncbi.nlm.nih.gov/blast/executables/blast + /. The best BLASTX hit based on query coverage, identity, similarity score, and description of each gene was filtered out using in-house pipeline.

### Organism annotation

The top BLASTX hit of each gene was studied and the organism name was extracted. The top 10 organisms were used for blastx hits. Prior to performing comparative genomes for newly isolated strains, the sequence was first re-annotated using the RAST server that is available via http://rast.nmpdr.org/rast.cgi. The genome was collected from an annotated server.

### Assign annotation for predicted genes

The predicted genes were annotated against UniProt and other databases; 5652 genes were annotated using BLASTX searches against Uniprot database (ftp://ftp.ncbi.nlm.nih.gov/blast/executables/blast+/).

## Genomic DNA annotation from RAST server perspective

From de novo DNA assembly, genome annotation was performed for BNPI-92 and other closely related strains using RAST server [30] which is available at http://rast.nmpdr.org/rast.cgi with the default settings. Briefly, assembly of *Bacillus* sp. BNPI-92 genomic DNA was computed into EzTaxone server (https://www.ezbiocloud.net/tools) to extract 16SrRNA. Five complete genomic DNA sequences of closely related bacterial strains (*B. cereus* ATCC 14579^T^ (AE016877), *B. paranthracis* Mn5^T^ (MACE01000012), *B. thuringiensis* ATCC 10792^T^ (ACNF01000156), *B. anthracis* str. Amen^T^ (AE016879), and *B. wiedmannii* FSL W8-0169^T^ (LOBC01000053) which related to *Bacillus* sp. BNPI-92 were collected from EzBioCloud Genome database (http://www.ezbiocloud.net/) based on their 16SrRNA gene sequence similarity. These strains were then re-annotated using the RAST server available via http://rast.theseed.org/FIG/rast.cgi. From annotation, functional genes, location of genes, and metabolic pathways for PHA were predicted.

## Comparatzive genomic DNA analysis

### Comparative genomic analysis using Ortho Venn^2^

A re-annotated protein sequence corresponding to these six strains was collected from the RAST server in the form of a fasta file. These protein sequences were uploaded into the OrthoVenn2 [18] server that is available via https://orthovenn2.bioinfotoolkits.net/home. The Occurrence table, Venn diagram, that displays the distribution of shared orthologous clusters among these six *Bacillus* strains keyword and cluster ID search for specific clusters, counts of clusters in each genome, and pairwise heatmap of overlapping gene clusters for these strains were performed at threshold E-value of 1e^−^5 and inflation value of 1.5.

### Gene ontology

The Gene Ontology (GO) terms such as biological process (BP), cellular component (CC), and molecular function (MF) were mapped using the in-house pipeline. Gene ontology (GO) was predicted from the output of the metabolic pathway and assigned for corresponding strains.

### Comparative genome analysis using OAT software

A 16SrRNA gene sequence was extracted from WGS using EzBioCloud available via https://www.ezbiocloud.net/identify and phylogenetic tree performed using Mega 7.0.9 program. The BNPI-92 16SrRNA gene sequence was deposited in the NCBI database with OP329213 accession number. Re-annotated genomes (Fasta format) of these six closely related and *Bacillus* sp. BNPI-92 strains were computed into the OAT software (OrthoANI Tool version 0.93.1). Comparative genome similarities for these strains were calculated using Ortho ANI, original ANI, GGDC 2.1, and *is*DDH tools. Heatmap with values and unweighted pair group method with arithmetic mean (UPGMA) trees between *Bacillus* sp. BNPI-92 and these closely related strains were calculated and generated [17].

### ANI calculator

The ANI between two genomic datasets of *Bacillus* sp. BNPI-92 and corresponding these five strains were calculated using ANI calculator available at http://enve-omics.ce.gatech.edu/ani/ for both best hits (one-way ANI) and reciprocal best hits (two-way ANI) [31]. If ANI between two genomic datasets were > 95%, the organisms would be considered as the same species. If its ANI were below 75%, it would be rejected [32].

## TYPE strains genome server for closely related and annotated strains

The genome sequence data were uploaded to the Type (Strain) Genome Server (TYGS). It is a free bioinformatics platform available via https://tygs.dsmz.de, for a whole genome-based taxonomic analysis [33]. In brief, the TYGS analysis was subdivided into the following steps:

### Determination of closely related type strains

The 16S rRNA gene sequences were extracted from the user genomes using RNAmmer [34] and each sequence was subsequently BLASTed [35] against the 16S rRNA gene sequence of each of the currently 9844 type strains available in the TYGS database. This was used as a proxy to find the best 50 matching type strains (according to the bit score) for each user genome and to subsequently calculate precise distances using the Genome BLAST Distance Phylogeny approach (GBDP) under the algorithm “coverage” and distance formula d_5_ [36]. The distances were finally used to determine the 10 closest type strain genomes for each of the user genomes.

### Pairwise comparison of genome sequences

All pairwise comparisons among the set of genomes were conducted using GBDP and intergenomic distances inferred under the algorithm “trimming” and distance formula d_5_ [36]. 100 distance replicates were calculated each. Digital DDH values and confidence intervals were calculated using the recommended settings of the GGDC2.1 [36]. Phylogenetic inference: The resulting intergenomic distances were used to infer a balanced minimum evolution tree with branch support via FASTME 2.1.4 including SPR post-processing [37]. Branch support was inferred from 100 pseudo-bootstrap replicates each. The trees were rooted at the midpoint [38] and visualized with PhyD3 [39].

Type-based species and subspecies clustering: The type-based species clustering using a 70% *is*DDH radius around each of the 10 type strains was done as previously applied [40]. Subspecies clustering was done using a 79% *is*DDH threshold as previously introduced [41].

## Metabolic pathways comparison using Heat map tool

RAST annotated protein sequences were computed into the KEGG-KAAS pathway. KEGG metabolic pathways were performed for complete annotated protein sequences of *Bacillus* sp. BNPI-92 and other closely related *Bacillus* strains using KAAS job request (BBH method). It was available via https://www.genome.jp/kaas-bin/kaas_main. The KEGG numbers [42] were collected for closely related strains. These KEGG numeric data were computed into a minPath server that is available via http://omics.informatics.indiana.edu/MinPath/run.php to search for the conserved metabolic pathway. The minPath results were collected from “Results in html link”, copied, and transferred into excel. The completeness of the metabolic pathway was checked and its percentage of completeness of metabolic pathway was calculated as Families annotated / Families involved × 100%. Finally, Heatmap [43] was computed into the ClustVis server available at https://biit.cs.ut.ee/clustvis/ and performed for the corresponding secondary metabolite.

## Ribosomal multilocus sequence typing (rMLST)

The rMLST were conducted for *Bacillus* sp. BNPI-92. It was performed using an online tool that is available at https://pubmlst.org/species-id.

## Results

### De novo assembly and scaffold sorting

The lowest contigs (≥ 0 bp) (156) and highest (7441) were obtained from Velvet and SPAdes, respectively (Table 1) against *BNPI*-92 strain after these de novo assembly was performed. As it was revealed in the Table 1, equal amount of GC % (35) was recorded for genomic DNA of BNPI-92 strain against ABySS and Velvet tools. The lowest (116,621) minimum contig length which required to cover 50% of the assembled genome sequence (N_50_) (Table 1) was obtained from ABySS tools. However, the highest (202,477) N_50_ was documented against SPAdes and followed by Velvet (200,267) tool. The default k-mer sizes were used for SPAdes assembly (Table 1). The complete assembly statistics made using QUAST 4.0 and BUSCO 2.0 were obtained for BNPI-92 strain (Table 1). As indicated in Table 1, other various values for the GC (%), N_50_, N_75_, L_50_, L_75_, total BUSCO groups searched, complete BUSCOs (C), complete and single-copy BUSCOs (S), complete and duplicated BUSCOs (D), fragmented BUSCOs (F), and missing BUSCOs (M) were obtained against ABySS, SPAdes, and Velvet tools.

**Table 1:** Assembly statistics QUAST 4.0 and BUSCO 2.0

S.N	Assembly statistics	ABySS	SPAdes	Velvet
1	contigs (≥ 0 bp)	376	7441	156
2	contigs (≥ 500 bp)	150	864	116
3	contigs (≥ 1000 bp)	124	122	96
4	contigs (≥ 5000 bp)	89	52	52
5	contigs (≥ 10,000 bp)	75	43	39
6	contigs (≥ 25,000 bp)	56	34	32
7	contigs (≥ 50,000 bp)	36	28	26
8	Average no. of contigs/scaffold (bp)	129.43	1226.285	73.85714
9	Total length (≥ 0 bp)	5,822,949	8,148,351	5,527,513
10	Total length (≥ 500 bp)	5,777,667	6,315,806	5,515,700
11	Total length (≥ 1000 bp)	5,759,070	5,832,559	5,501,478
12	Total length (≥ 5000 bp)	5,673,015	5,722,474	5,391,047
13	Total length (≥ 10,000 bp)	5,579,289	5,653,765	5,295,275
14	Total length (≥ 25,000 bp)	5,292,792	5,492,325	5,185,005
15	Total length (≥ 50,000 bp)	4,524,924	5,271,241	4,982,387
16	Largest contigs	537,434	732,848	698,773
17	GC (%)	35	38	35
18	N_50_	116,621	202,477	200,267
19	N_75_	61,283	94,390	137,847
20	L_50_	15	9	8
21	L_75_	33	21	16
22	Ns per 100 kbp	13.57	0	0
23	Total BUSCO groups searched	148	148	148
24	Complete BUSCOs (C)	147	147	147
25	Complete and single-copy BUSCOs (S)	144	143	144
26	Complete and duplicated BUSCOs (D)	3	4	3
27	Fragmented BUSCOs (F)	0	0	0
28	Missing BUSCOs (M)	1	1	1

## Fastq quality

Fastq quality checking and filtering involves checking of quality parameters for the sequences obtained from the sequencer.

## Gene prediction and genome coverage

### Matching with UniProt database using BLASTX program

We predicted genes from the Velvet assembled contigs using Glimmer software [44]. In this WGS analysis, 5719 genes were predicted in the assembly. These predicted genes were annotated and found to be 5652 genes (Additional File 1: Table S1). The genome coverage for *Bacillus* sp. BNPI-92 was predicted to be 123X for raw read summary and 71X for cleaned reads statistics. However, for raw read and cleaned reads statistics the genome coverage for *Bacillus* sp. BNPI-92 strain was 193X.

## Gene annotation against UniProt databases

### Organism annotation

The top 10 BLASTX hits of each gene indicated that the recent isolate is allied with *Bacillus* strains (Additional file 1: Fig. S1). As shown in Additional file 1: Fig. S1, *Bacillus sp. KbaL1* is found to be the majority of the top BLASTX hit strain (> 3000) and followed by *Bacillus* sp. YF23 (485) and *B. cereus* (339). However, the lowest number of hits (93) was predicted between the recent isolate and *B. cereus* Rock 1–15 (Additional file 1: Fig. S1). However, annotation and comparative genomic DNA analysis indicated that *Bacillus* sp. BNPI-92 strain was similar to *B. cereus* ATCC 14579^T^.

## The RAST server: rapid annotations using subsystems technology and genomic features of annotated *Bacillus* sp. BNPI-92

**Fig. 1:**
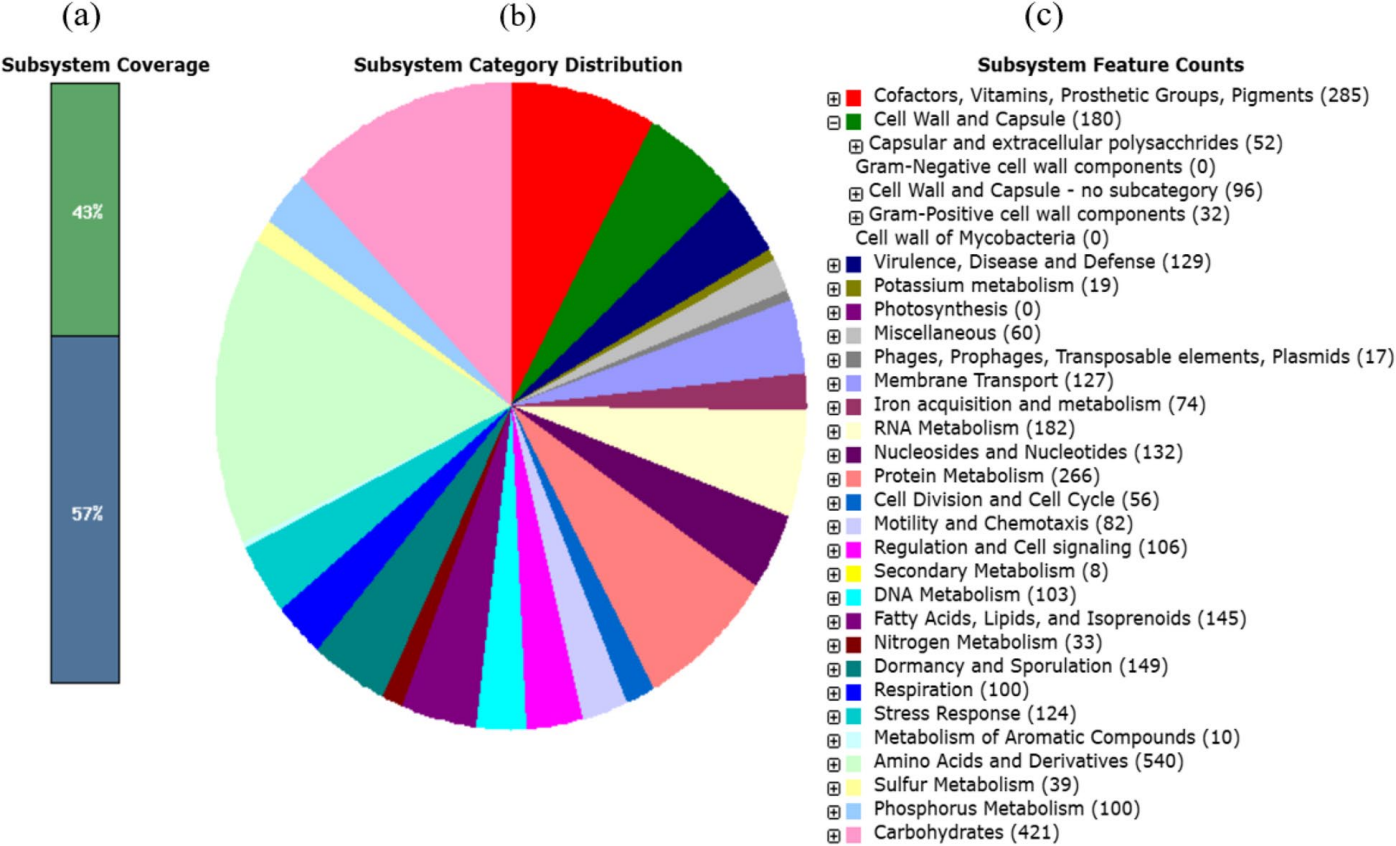
Graphical representation of annotated genome assembly of BNPI-92 based on RAST server. Genome assembly (genes) connected to subsystems and their distribution in different categories. **a** Subsystem coverage, **b** subsystem category of percentage distribution, and **c** subsystem feature count that expandable down to the specific gene with their respective role (see secondary metabolism). This online tool is available at http://rast.theseed.org/FIG/rast.cgi

A 35.1% GC content was generated for *Bacillus* sp. BNPI-92’s genome assembly from RAST annotation. Various genomic features were recorded against BNPI-92 and other closely related strains (Table 2). As illustrated in Table 2, the highest sequence size (5,527,513), number of contigs (156), and number of subsystems (481) were obtained from *Bacillus* sp. BNPI-92 A *Bacillus* sp. BNPI-92 strain genome annotation is also graphically depicted in Fig. 1.

**Table 2:** Features of annotated genomic DNA for BNPI-92 and its allied reference strains

No	Statistics	As uploaded and after splitting into scaffolds					
		*Bacillus* sp. BNPI-92	*Bacillus cereus* ATCC 14579	*Bacillus thuringiensis*	*Bacillus paranthracis* Mn5	*Bacillus wiedmannii* FSL W8-0169	*Bacillus anthracis* Ames
1	Sequence size	5,527,513	4,190,892	936,205	5,513,239	3,763,655	5,227,293
2	Number of coding sequence	5679	4396	896	5675	4014	5642
3	Number of contigs	156	2	1	91	39	1
4	GC content (%)	35.1	35.0	36.9	35.2	35.2	35.4
5	Shortest contig size	201	15,274	35.4	204	251	5,227,293
6	Median sequence size	1880	5,411,809	77,112	17,468	65,251	5,227,293
7	Mean sequence size	35,432.8	2,713,541.5	5,237,682	60,585.0	96,504.0	5,227,293
8	Longest contig size	698,773	541,180.9	2,657,397.0	803,208	583,560	5,227,293
9	N50 value	200,267	5,411,809	5,237,682	135,274	145,774	1
10	L50 value	8	1	1	9	7	1
11	Number of RNA	57	102	100	19	29	117
12	Number of subsystems	481	275	333	480	287	360

As shown in Fig. 1a, subsystem coverage (bar chart) and non-subsystem coverage were found to be 43 and 57%, respectively. It was predicted that there is total coverage of 2408 subsystems and 3271 non-subsystems for *Bacillus* sp. BNPI-92 strain. In subsystem coverage, the hypothetical and non-hypothetical gene sizes are 125 and 2283, respectively (Fig. 1a). The percentage distribution of subsystem features was depicted in graphical representation (Fig. 1b). The highest percentage of subsystem features was detected for amino acids and derivatives and followed by carbohydrate metabolic features. However, the lowest percentage was detected for phages, prophages, transposable elements, and plasmids subsystem feature (Fig. 1c) when expanded down to the specific gene feature. As it was shown in Fig. 1c, a central carbohydrate metabolisms (123), aminosugars (10), di- and oligosaccharides (55), one-carbon metabolism (56), organic acid (44), fermentation (e.g., acetyl-CoA to butyrate) (66), sugar alcohol (17), polysaccharides (7), and monosaccharides (48) were predicted in carbohydrate metabolic subsystems (421).

## Genome mapping for *Bacillus* sp. BNPI-92 strain

**Fig. 2:**
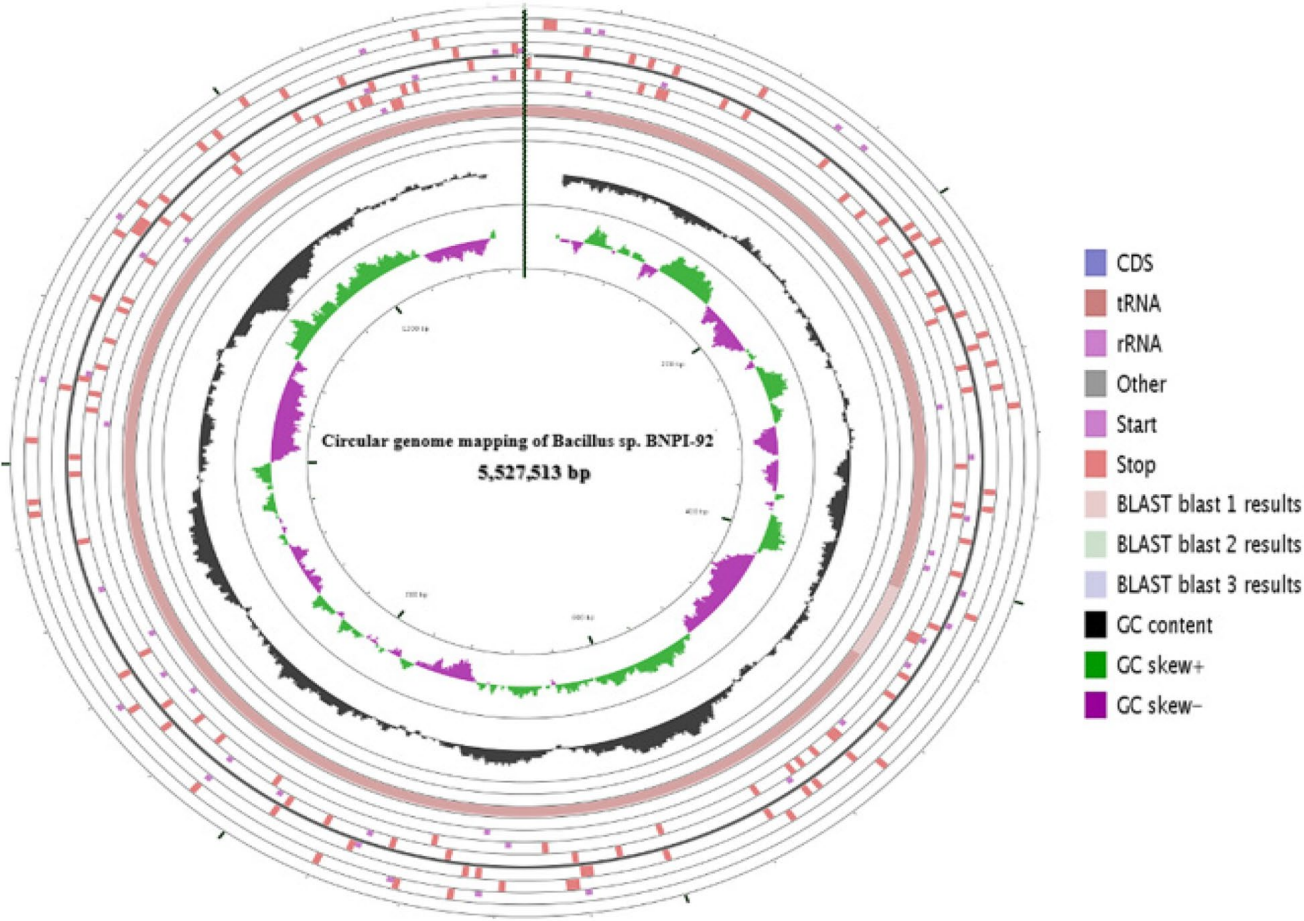
Graphic circular genome mapping for *Bacillus* sp. BNPI-92 that produced PHA polymers and obtained from area of plastic wastes accumulation. This genome map is visualized by CGview.ca that available at http://stothard.afns.ualberta.ca/cgview_server/

Circular genome mapping for *Bacillus* sp. BNPI-92 genomic DNA was obtained using CGView online server that is available via http://stothard.afns.ualberta.ca/cgview_server/. GC content, open reading frame, GC skrew ^(±)^, rRNA, starting codon, stop codon, and CDC were predicted on the genome mapping (Fig. 2). In this result, it was found that assembled and annotated genomic DNA of BNPI-92 strain has 5,527,513 bp gene size, 35.5 mol% G + C content, 481 number of subsystems, 8L_50_ and 200, 267 N_50_ (Fig. 2). From RAST server output, *Bacillus* sp. BNPI-92 genome is predicted to contain 156 contigs, 5679 amino acid coding sequence, and 57 numbers of RNA (Fig. 2).

## Comparative genomic analysis using Ortho Venn^2^ and pair wise

**Fig. 3:**
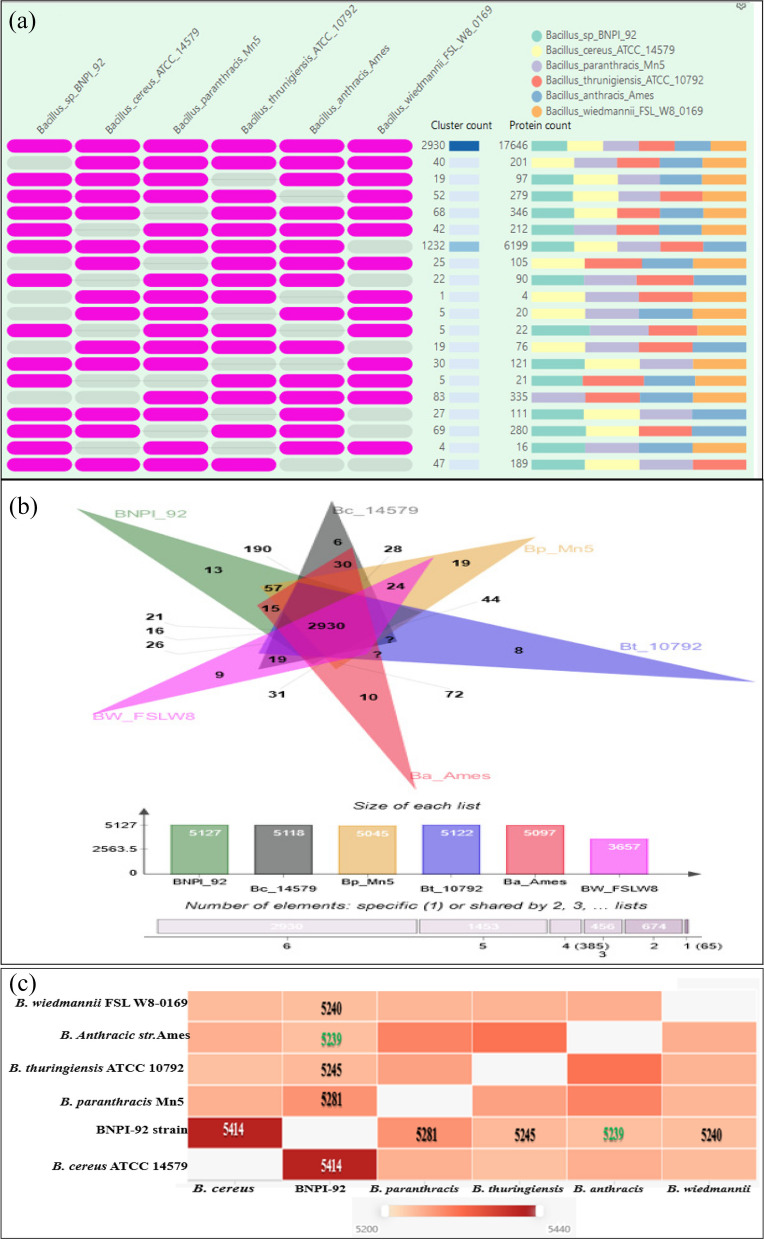
Ortho Venn analysis using online tools that available at available via https://orthovenn2.bioinfotoolkits.net/task/create. **a** The occurrence table contains multiple groups of gene cluster (the pattern to the left which indicates the species are in the clusters) such as cluster count (number of gene clusters shared between species) and protein count (number of protein members in the shared cluster for these strains). Row indicates orthologous gene cluster for multiple species that summarized as a cell graph and column indicates different closely related bacterial species. The occurrence table with deep purple color bar represented the pattern of shared multiple orthologous gene cluster among *Bacillus* sp. BNPI-92 and other closely related bacterial strains whereas gray color bars indicate the absence of gene cluster in these strains (Fig. 5a). **b** OrthoVenn diagram graphic tools used for comparing a protein sequence of PHA-producing BNPI-92 strain with other five closely related strains. **c** Similarity matrix for pairwise protein sequence comparison for heatmap that shows the orthologs cluster between *Bacillus* sp. BNPI-92 and other closely related strains of protein sequence. This heatmap [43] was computed into ClustVis server that is available at https://biit.cs.ut.ee/clustvis/

In this study, after comparative genomics had been performed for PHA-producing *Bacillus* sp. BNPI-92 (VXJL00000000) and other five closely related bacterial strains (*B. cereus* ATCC 14579^T^ (AE016877), *B. paranthracis* Mn5^T^ (MACE01000012), *B. thuringiensis* ATCC 10792^T^ (ACNF01000156), *B. antrics* Amen^T^ (AE016879)), various results were obtained (Fig. 3a, Additional File 1: Table S2). The first pattern summarized in the cell graph displayed in the occurrence table was gene clusters (orthologous cluster group) (Fig. 3a, Additional File 1: Table S2). The second pattern represents cluster counts and the third pattern of stacked bars displayed at the right place represents a collective number of protein sequences present in the cluster group for corresponding strains (Additional File 1: Table S2).

As displayed in Venn diagram graphics (Fig. 3b), *Bacillus* sp. BNPI-92 strain shared a common protein sequence with these six aligned and closely related *Bacillus* strains. These common distributions of protein sequences (the protein encoded with orthologous gene clusters) among the first six bacterial strains were shown in the occurrence table (Fig. 3a). The deep purple colors represented cluster genes for respective reference strains and PHA-producing *Bacillus* sp. BNPI-92 strain. It was observed that 2930 gene clusters were shared among these closely related strains (Fig. 3a). A number of orthologous protein-coding gene clusters shared by these strains were also represented in Venn diagram graphics (Fig. 3b).

As shown in the Venn diagram, overlapping gene clusters were predicted between *Bacillus* sp. BNPI-92 strains and these closely related strains. The minimum overlapping gene clusters (16 gene clusters) were predicted between BNPI-92 and *B. wiedmannii* FSL W8 0169 strains (Fig. 3b). However, the maximum overlapping gene clusters (190 gene clusters) were detected between BNPI-92 and *B. cereus* ATCC 14570^T^. In the recent study, it was realized that newly PHA-producing isolates, *Bacillus* sp. BNPI-92, did not share 13 gene clusters with these allied strains (Fig. 3b).

The pairwise heatmap was obtained against *Bacillus* sp.BNPI-92 and other closely related strains (Fig. 3c). The minimum thresholds of overlapping gene clusters were detected between *Bacillus* sp. BNPI-92 and *B. anthracis* Ames (5239) (Fig. 3c). However, as indicated in Fig. 3c, the maximum overlapping gene clusters threshold has been detected between *Bacillus* sp. BNPI-92 and *B. cereus* ATCC 14579^T^ (5414) with a deep red color gradient (Fig. 3c).

## Gene ontology from perspective of Ortho Venn.^2^

A core genome belonging to PHA-producing BNPI-92 and these closely related strains were predicted in Fig. 3b using Venn diagram graphics. Biological processes, molecular functions, and cellular components gene ontology (GO) with corresponding functional gene clusters were predicted (Additional File 1: Table S3). Few of these gene clusters were cluster 87, 93, 191, 273, 300, 351, 380, 390, 563, and 1873 in GO terms. Secondary metabolite encoding gene clusters were predicted. Few of these gene clusters were glucose metabolic (GO:0006006), carbohydrate metabolic (GO:0005975), pyruvate metabolic (GO:0006090), 3-hydroxybutyrate dehydrogenase (GO: 0003858, cluster2158), glycerol-3-phosphate metabolic (GO:0006072), butyrate metabolic (GO:0019605, cluster 380), lipid metabolic (GO:0006629), acyl-CoA metabolic (GO:0006637), and organic acid metabolic (GO:0006082). Multiple gene clusters (cluster23, 54, 55, 108, 147, 149, 157, etc.) for sporulation (GO:0030435), were similarly predicted in these genomes.

**Fig. 4:**
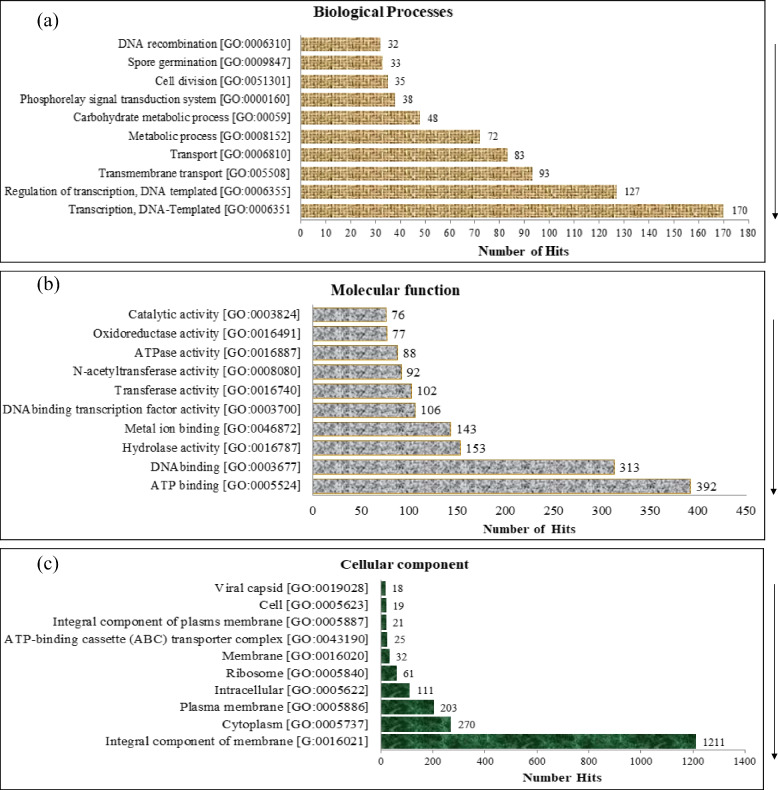
**a** Top 10 terms in biological process category from GO annotation of BNPI-92 strain. **b** Top 10 terms in molecular function category from GO annotation of BNPI-92 strain. **c** Top cellular component category from GO annotation of BNPI-92 strain

In the present, using UniPort in-house pipeline that is available at http://www.geneontology.org GO was predicted for BNPI-92 strain whole-genome sequence (Additional File 1: Table S2). Using the same pipeline, top 10 terms in each category are shown in Fig. 4a–c.

### Biological process

Certain protein-coding genes were identified for BNPI-92 strain in terms of biological process (Fig. 4a). The highest number of hits (170) was predicted for transcription DNA template followed by regulation of transcription DNA template (Fig. 4a). However, the lowest number of hits (32) was observed for DNA recombination (Fig. 4a). In the current BNPI-92 strain’s genomic analysis, carbohydrate metabolic process and metabolic process were few predicted Gene Ontology terms in biological process category (Fig. 4a). It was found that 475 number of terms were predicted (log (^p−value^) for biological process category including transcription DNA template, regulation of transcription DNA template, and DNA recombination had been few biological GO predicted.

### Molecular functions

Various molecular functions GO terms were obtained against BNPI-92 strains that were selected as a potential PHA-producing bacterial isolate (Fig. 4b). A total of 802 numbers of terms (Fig. 4b) were predicted for BNPI-92 strain when molecular function genome annotation had been performed. ATP binding (GO:0005524), catalytic activity (GO:0003824), and DNA binding (GO:0003677) were few molecular functions GO terms detected.

### Cellular component

In this result, cellular component GO category (69) was predicted for BNPI-92 strain which is a less number of terms (Fig. 4c). It was predicted that the integral component of membrane (GO:00016021) category is the highest number of hits (with highest enrichment score) in cellular component GO term and followed by cytoplasm GO (GO:0005737) category (Fig. 4c).

## Comparative genome analysis for *Bacillus* sp. BNPI-92 using OAT tools

**Fig. 5:**
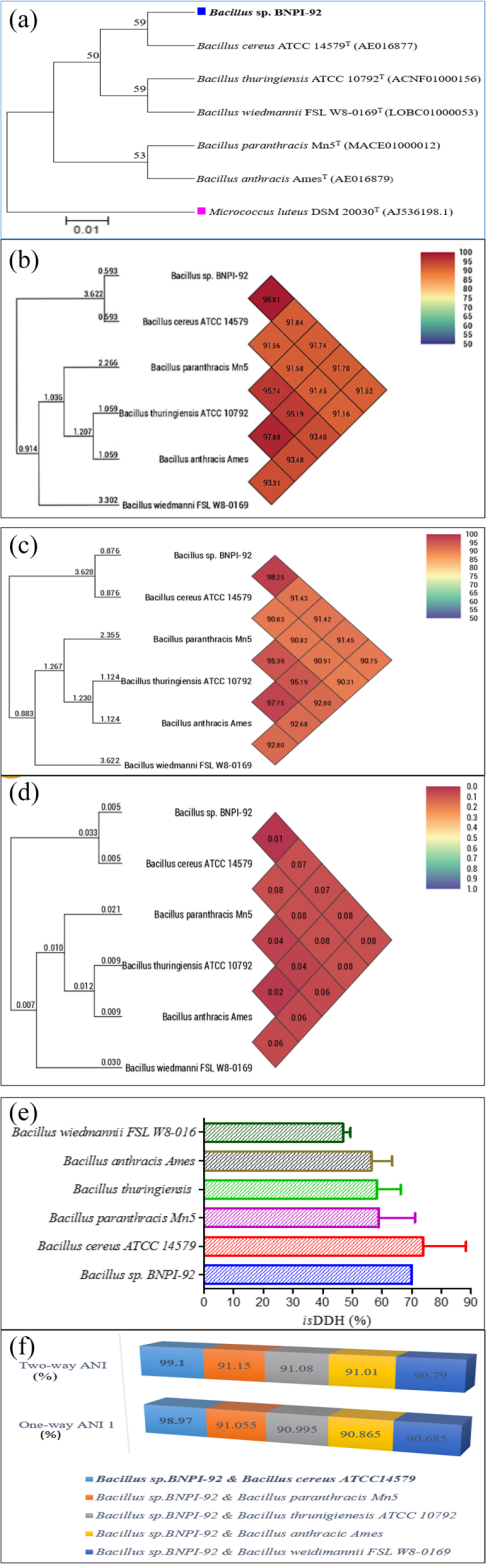
** a** Phylogenetic tree and evolutionary relationships of taxa for Bacillus sp. BNPI-92 and the other closely related strains. The evolutionary history was inferred using the neighbor-joining method [45]. The optimal tree with the sum of branch length = 0.18016536 is shown. Evolutionary distances were calculated by neighbor-joining and based on 1000 bootstrap replication of confidence values (percentage of 1000 replication). Bar, 0.05 substitutions per nucleotide position [46]. The evolutionary distances were computed by using p-distance method [47]. The analysis involved six nucleotide sequences. All positions containing gaps and missing data were eliminated. Finally, evolutionary analyses were performed with MEGA7.0.9 software [48]. Micrococcus luteus DSM 20030 T (AJ536198.1) was designated and used as outgroup in the analyses; other related sequences were obtained from EzTaxon-e server and annotated using RAST server before tree construction **b** UPGMA dendrograms heatmap for OrthoANI [17], **c** UPGMA dendrograms heatmap for OriginalANI [17] and **d** UPGMA dendrograms heatmap for genome to genome distance calculator (GGDC) [17]. **e** In silicon DDH ( is DDH) for Bacillus sp. BNPI-92 and closely related strains. In silicon DDH analysis was performed using online tool that is available at https://tygs.dsmz.de/and Fig. 8e constructed using GraphPad Software. **f** Average nucleotide identity (ANI) (%) between pairs of Bacillus sp. BNPI and other five strains. ANI values of ≥ 96% and is DDH values of ≥ 70% consistently grouped genomes originating from strains of the same species together. It was performed according to Goris et al . [31] and using online tools that are available at http://enve-omics.ce.gatech.edu/ani/

Our result indicated that the sequences were aligned using ClustalW and found that (Fig. 5a) BNPI-92 strain shows 100% sequence similarity with *B. cereus* ATCC 14579^T^ and followed by *B. anthracis* Ames and *B. paranthracis* Mn5 strains with each 99.93% sequence sharing.

Orthologous Average Nucleotide Identity (OAT) tools such as OrthoANI, Original ANI, and GGDC used to predict sequence similarity between BNPI-92 and other allied stains. BNPI-92 strain (VXJL00000000) showed more than 91% genome sequence similarity with these strains (Fig. 5b, c) against OrthoANI. Using the OAT tool, it was realized that BNPI-92 strain genomic DNA was closest to *B. cereus* ATCC 14579^T^ with 98.81% OrthoANI (Fig. 5b) followed by *B. paranthracis* Mn5 (91.84%, OrthoANI), *B. thuringiensis* ATCC 10792 (91.74%, OrthoANI), *B. anthracis* Ames (91.70%, OrthoANI), and *B. wiedmannii* FSL W8-0169 (91.52%, OrthoANI) (Fig. 5b).

OrthoANI and original ANI showed a strong relation. As it was displayed in Fig. 5b and c, OrthANI values (91.52–98.81%) between BNPI-92 and other closely related strains were higher than original ANI values (90.75–98.25%) recorded for these strains. The minimum OriginalANI (90.75%) was calculated between BNPI-92 and *B. wiedmannii* FSL W8 0169^T^ strains whereas the maximum OriginalANI (98.25%) (Fig. 5b) was calculated between BNPI-92 and *B. cereus* ATCC 14579^T^ strains.

*Digital*/*in silicon* DNA–DNA hybridization (*d/is*DDH) was performed for BNPI-92 and these (5) closely related strains (Fig. 5e). The minimum average sequence similarity for *is*DDH was predicted between BNPI-92 and *B. wiedmannii* FSL W8-0169 (46.8%) strain (Fig. 5e). However, the maximum average sequence similarity for *is*DDH value was predicted between BNPI-92 and *B. cereus* ATCC 14579^T^ (73.73%) strain which is ≥ 70% (Fig. 5e).

ANI between BNPI-92 and other closely related pair genomic database were determined using ANI calculator available at http://enve-omics.ce.gatech.edu/ani/ for both best hits (one-way ANI) and reciprocal best hits (two-way ANI). It was observed that the average nucleotide identity (ANI) between BNPI-92 and *B. cereus* ATCC 14579^T^ strain was 99.10% (Fig. 5f). And the maximum ANI has been calculated as it displays in Fig. 5f between BNPI-92 and *B. cereus* ATCC 14579^T^ (99.1%) strain.

## TYPE strains genome server for closely related and annotated strains

**Fig. 6:**
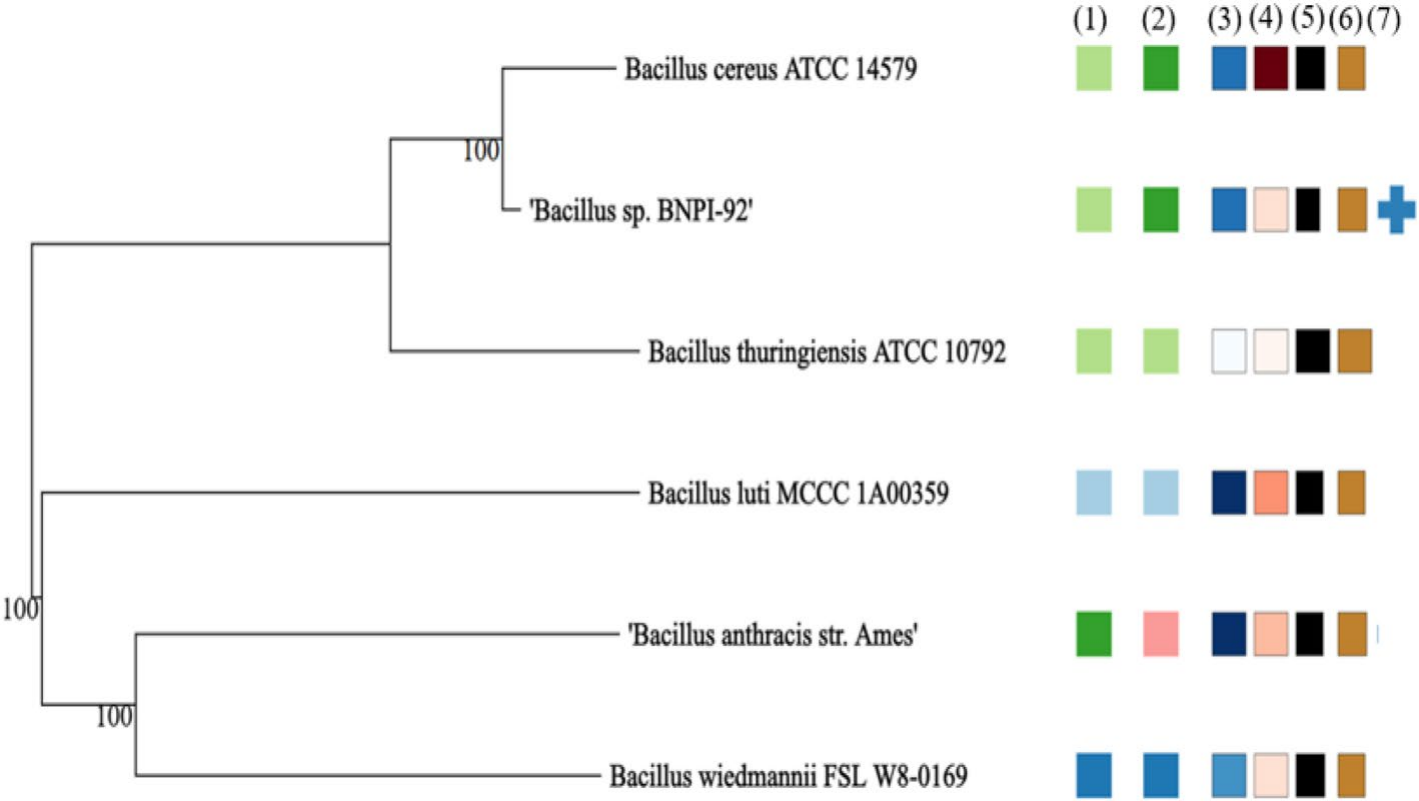
GBDP phylogeny based on genome data and TYGS result for PHA-producing bacterial isolate data set. Tree inferred with Fast ME 2.1.6.1 [37] from GBDP distances calculated from genome sequences of *Bacillus* sp. BNPI-92 and other closely related *Bacillus* strains. The branch lengths are scaled in terms of GBDP distance formula d_5_. The numbers above branches are GBDP pseudo-bootstrap support values > 60% from 100 replications, with average branch support of 99.9%. The tree was rooted at the midpoint [38]. A labeled and colored box are annotated by affiliation and corresponding to (1) *is*DDH species clusters, (2) *is*DDH sub-species clusters, (3) percent GC (34.8–35.3%), (4) delta statistics (0.1–0.2), (5) genome size (4,614,627–6,234,842 bp), (6) protein count (5255–6243), and (7) user strain (query sequence)

Based on TYGS (Type (Strain) Genome Server) analysis, BNPI-92 was identical as *B. cereus* ATCC 14579^T^ strain at species and subspecies level (Fig. 6) when this stain had been compared with other related stains. The GC% contents were recorded between 34.8 and 35.3% for all strains. A protein-coding region (protein count) (5255–6243) (Fig. 6) for BNPI-92 and these related strains were predicted in Fig. 6 along with Delta statistics and genome size in the range of 0.1–0.2 and 4,614,627–6,234,842 bp, respectively.

## Metabolic pathway comparison using heatmap

BNPI-92 contains certain gene-encoded metabolic pathway (Additional file 1: Fig. S2) which is related to the other allied stains. However, the level of gene expressions was various among these metabolic pathways (Additional file 1: Fig. S2) for respective strains. In Fig. S2 (Additional file 1: Fig. S2), BNPI-92 shows very fewer levels of gene expression for various metabolic pathways gene clusters. A highly expressed gene for biosynthesis of vancomycin group antibiotics and thiamine metabolic pathways were detected for BNPI-92 isolates. However, very fewer expressions were detected for focal adhesion (0.8%), epithelial cell signaling in Helicobacter pylori infection (1.82%), phosphotransferase system (PTS) (10%), methane metabolite (12.24%), gamma-hexachlorocyclohexane degradation (13.64%), and butanoate metabolite (34.33%) pathways (Additional file 1: Fig. S2).

## Annotation overview and PHA biosynthesis gene organization in *Bacillus* sp.BNPI-92 strain

**Fig. 7:**
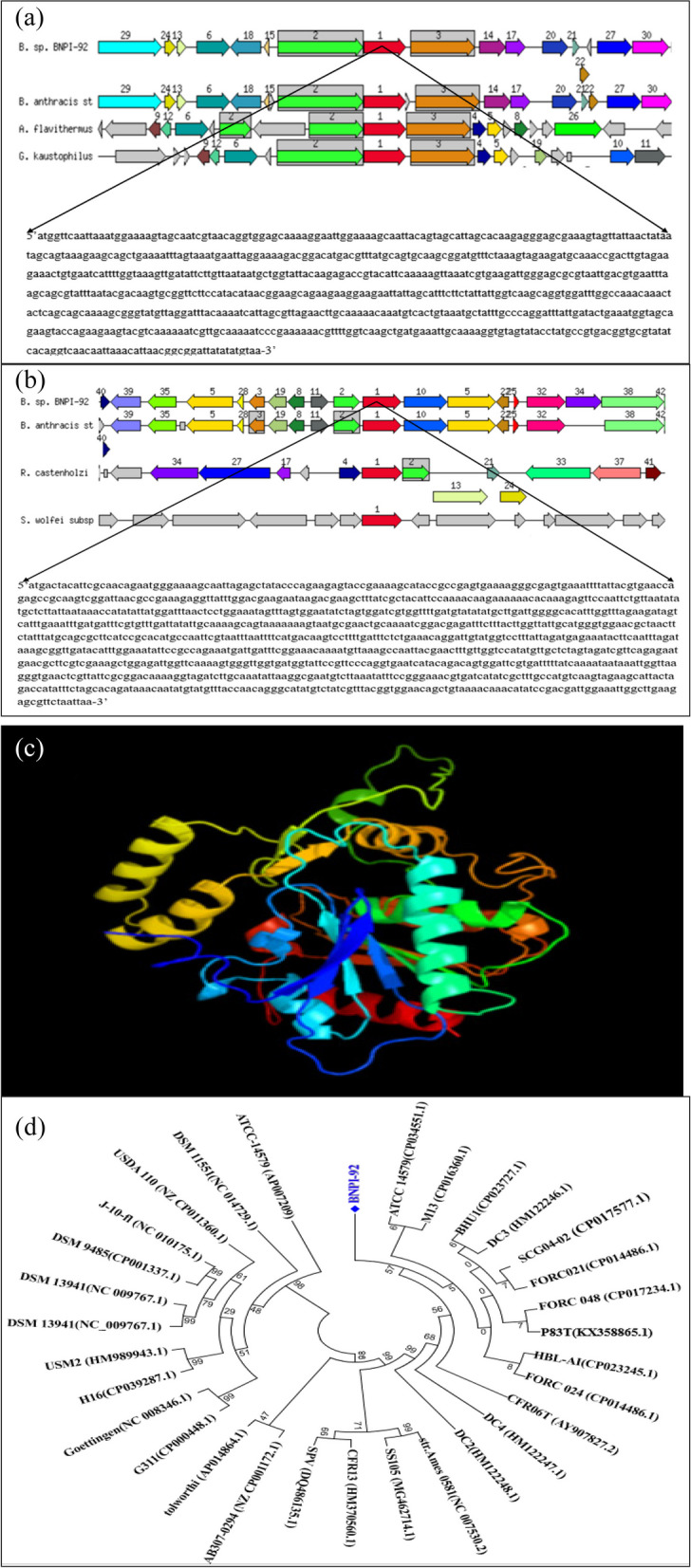
Annotation overview and schematic representation of secondary metabolite gene clusters for PHA biosynthesis and a comparative PHA genetic organization for *Bacillus* sp. BNPI-92 and other closely related strains from RAST server annotation for **a** and **b** using online tools that are available at http://rast.theseed.org/FIG/rast.cgi. A deep red colored arrow indicates a *phaC* gene that is supposed to encode protein used for PHA polymerization. **a** is a *phaA* gene or nucleotide sequences (744 bp) encoded by acetyl-CoA thiolase and **b** is a *phaC* gene or nucleotide sequence (1086 bp) encoded by polyhydroxyalkanoic acid synthase. It is a conserved nucleotide sequence. And *phaB* gene (arrow with number 2) encodes acetoacetyl-CoA reductase. *phaB* and *phaC* genes are located on the same operon. Note that *phaB* gene sequence is not shown. **c** Three-dimensional structure of *phaC* protein with 319 residues (residue of protein structure for *phaC* gene) and presumed for PHA biosynthesis in *Bacillus* sp. BNPI-92. It has 319 residues. Its 88% has been modeled with 100.0% confidence by the single highest scoring template. The given protein residue resembles class I polyhydroxybutyrate synthase that was derived from *Cupriavidus necator* in terms of its structure. The three-dimensional structure of protein was predicted using PHYRE2 which is an online tool that is available at http://www.sbg.bio.ic.ac.uk/phyre2/html/page.cgi?id=index (**d**). Evolutionary relationships for *Bacillus* sp. BNPI-92

PHA biosynthesis encoding genes were predicted in the RAST server. In this annotation, *phaA, phaB*, and *phaC* genes were detected for BNPI-92 strain (Fig. 7). As it displays in graphic Fig. 7a, *phaB* and *phaC* genes have been located on the same operon for BNPI-92 strain. Annotated overview of a chromosomal region of *phaC* gene for BNPI-92 strain was compared with certain similar bacterial strains (database) (Fig. 7a). And in this graphic representation, sets of genes with a similar sequence are grouped with the same number and color (Fig. 7a). The functional and focus gene always points to the right direction as shown in Fig. 7a. Genes whose relative position is conserved in other species are functionally coupled. In this genome annotation output, the graphic centered on the focus gene with a red color arrow and numbered as a 1 is *phaC* gene (1086 bp length) (Fig. 7b). The graphic situated on the focus gene with green and numbered as a 2 is *phaB/phbB* gene (744 bp length) (Fig. 7a). In this graphic representation, it was displayed that the *phaB* gene existed in both BNPI-92 and *B. anthracis* st. Ames strains (Fig. 7a).

**Fig. 8:**
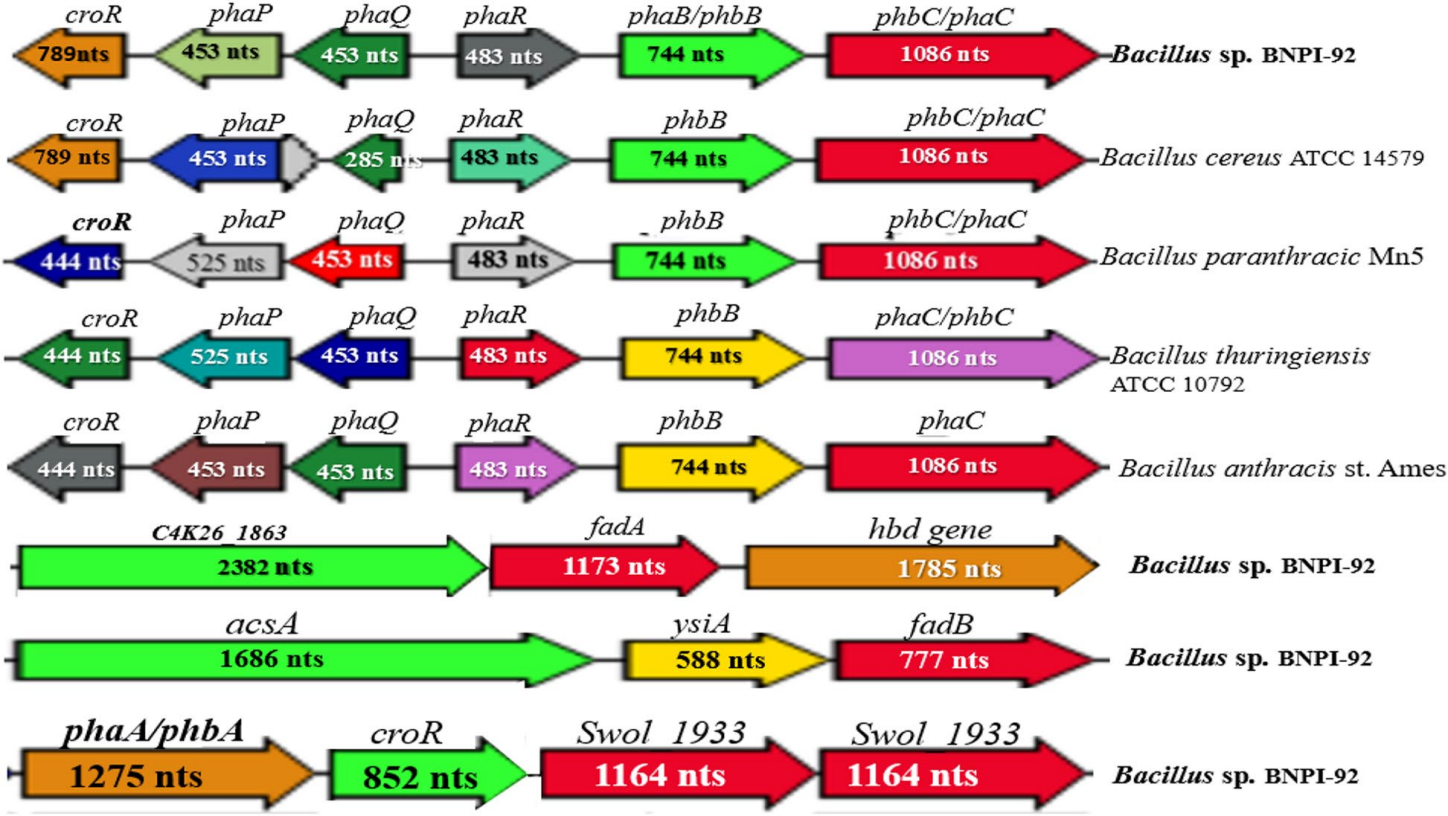
PHA biosynthesis promoting genetic organizations (gene loci) in Bacillus sp. BNPI-92 and other closely related type strains genome ( Bacillus cereus ATCC 14579 T (AE016877), Bacillus paranthracis Mn5 T (MACE01000012), Bacillus thuringiensis ATCC 10792 T (ACNF01000156), and Bacillus antrics Amen T (AE016879). These strains harbored PHA biosynthesis gene with nearly similar nucleotide sequence size (nts) such as croR (predicted for PHA metabolisms), PhaQ (a PHB-responsive repressor controlling expression of phaP ), phaP (predicted for phasin biosynthesis), phaA (suggested for acetyle fermentation), phaB (predicted for butyrate fermentation), phaR (suggested as class IV PHA synthase or polyhydroxyalkanoate synthesis repressor phaR ), and phaC (predicted as PHA polymerization). phaA was located on a separate gene and suggested for encoding 3-ketoacyl-CoA thiolase or acetyl-CoA acetyltransferase protein. phaJ [5] (encoding enoyl-CoA hydratase) and acsA gene [49] (encoding acetoacetyl-CoA synthetase) predicted for PHA biosynthesis were located on the same operon. Isolog butyryl-CoA dehydrogenase enzyme is a gene which is unidentified

It was found that *acetoacetyl-CoA reductase* was found to be *encoded* by *phaB* using RAST server pipeline. It was found that (Fig. 7a) *phaC* gene predicted in BNPI-92 strain shared (Fig. 7d) sequences similarity with other strains. Three-dimensional *phaC* crystal protein was obtained after its protein had been computed into PHYRE2 Protein Fold Recognition Server that is available at http://www.sbg.bio.ic.ac.uk/phyre2/html/page.cgi?id=index (Fig. 7c). And the 3D protein structures were constructed as displayed in Fig. 7c. Polyhydroxyalkanoate synthesis repressor *PhaR* gene sequence (Fig. 8) was detected in the BNPI-92 strain annotated genome sequence. As displayed in Fig. 8, *3-hydroxybutyryl-CoA dehydratase* enzyme was predicted and its gene was found to be *croR* gene (444 nts, 263 aa) based on RAST server annotation.

In Fig. 8, unidentified acetoacetyl-CoA synthetase [EC 6.2.1.16] (1686 nts, 562 aa) encoding gene was located on the same operon along with enoyl-CoA hydratase [EC:4.2.1.17] (777 nts, 259aa) encoding gene (*fadB)* in *B. cereus* BNPI-92 strain (Fig. 8). We realized that the presence of *phaA, phaB*, and *phaC* genes in our BNPI-92 strains suggested that this strain has a capability to synthesize short chain length (*sch*PHA) biopolymer. As illustrated in Fig. 8, *phaP* gene was predicted in BNPI-92 strains. However, its feature was not yet part of a subsystem using the RAST server pipeline. This gene may not be expressed despite existed on the operon along with *phaB, phaC*, and *phaR* genes. It was predicted that the *phaQ* gene encodes a P(3HB)-transcriptional regulator (*PhaQ*) protein as predicted from RAST server and KEGG metabolism pathways.

The evolutionary history of *phaC* protein was inferred using the neighbor-joining method [45]. The optimal tree with the sum of branch length = 1.29481050 is shown. The percentage of replicate trees in which the associated taxa clustered together in the bootstrap test (1000 replicates) was shown next to the branches [46]. The tree was drawn to scale, with branch lengths in the same units as those of the evolutionary distances used to infer the phylogenetic tree. The evolutionary distances were computed using the p-distance method [47] and are in the units of the number of base differences per site. The analysis involved 31 *phaC* protein or gene nucleotide sequences belonging to different corresponding bacterial strains mainly belonging to *Bacillus* strains. Codon positions included were 1^st^ + 2^nd^ + 3^rd^ + Noncoding. All positions containing gaps and missing data were eliminated. There was a total of 719 positions in the final dataset. Evolutionary analyses were performed in MEGA7 [48].

## Prediction of polyhydroxyalkanoates metabolic pathway

In RAST annotation, *enoyl-CoA hydratase* [EC 4.2.1.17] or 3-*hydroxyacyl-CoA dehydrogenase* [EC 1.1.1.35] encoding gene where located in the *B. cereus* BNPI-92 strain genome sequence along with *3-ketoacyl-CoA thiolase* [EC 2.3.1.16] or *acetyl-CoA acetyltransferase* [EC:2.3.1.9] (Fig. 8) and *butyryl-CoA dehydrogenase* (Fig. 8). However, in KEGG-KASS server, a gene encoding for *enoyl-CoA hydratase* and *3-hydroxyacyl-CoA dehydrogenase* were separately predicted as *fadB* and *fadN,* respectively*.* In Fig. 8, unidentified *acetoacetyl-CoA synthetase* [EC 6.2.1.16] (1686 nts, 562 aa) encoding gene was located on the same operon along with *enoyl-CoA hydratase* encoding gene (*fadB)* in *B. cereus* BNPI-92 strain (Fig. 8). PHA metabolic pathway was predicted by RAST server and KEGG-KASS server that available via https://www.genome.jp/kaas-bin/kaas_main. In these metabolic pathways, short-chain length of PHA (*sch*PHB) were predicted after a Fasta format of *B. cereus* BNPI-92 strain genome had been computed into KEGG-KASS server.

**Fig. 9:**
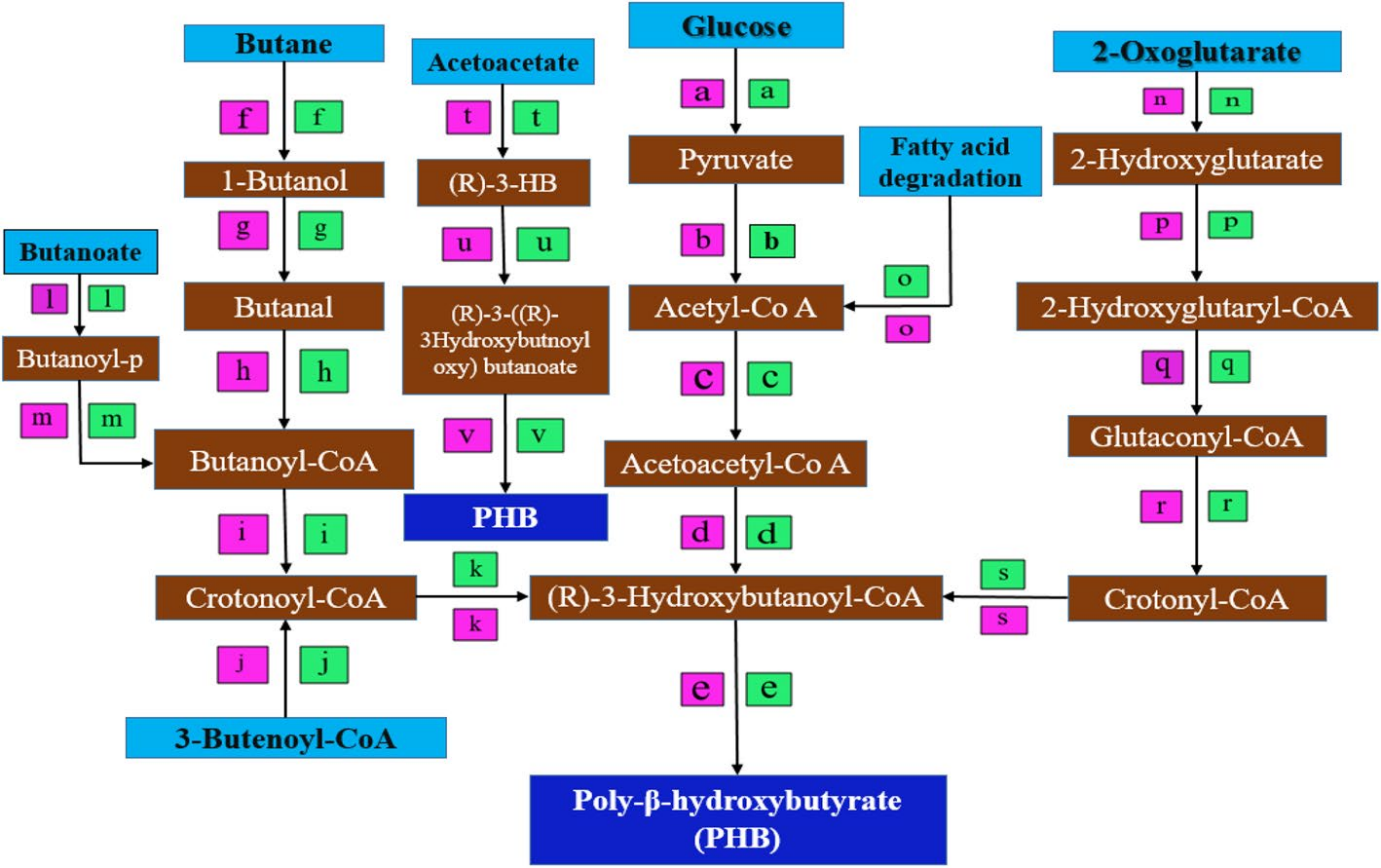
Metabolic pathway for PHA-producing Bacillus cereus BNPI-92 strain obtained from an area of plastic waste accumulation. A letter in green color box (a–v) indicates the most likely enzymes involved in metabolic activities. The letter in violet color box signifies possible gene involved in coding enzyme. List of enzymes: a not predicted in this study, b formate C-acetyltransferase [EC:2.3.1.54] (transferases), c acetyl-CoA C-acetyltransferase [EC:2.3.1.9] (transferases), d acetoacetyl-CoA reductase [EC:1.1.1.36] (oxidoreductases), e polyhydroxyalkanoate synthase subunit phaC [EC:2.3.1.-] (transferases), f butane monooxygenase alpha subunit [EC:1.14.13.230] (oxidoreductases), g butanol dehydrogenase [EC:1.1.1.-] (oxidoreductases), h acetaldehyde dehydrogenase (acetylating) [EC:1.2.1.10] (oxidoreductases), i butyryl-CoA dehydrogenase [EC:1.3.8.1] (oxidoreductases), k 3-hydroxybutyryl-CoA dehydratase/vinylacetyl-CoA-Delta-isomerase [EC:4.2.1.1205.3.3.3] (lyases), l butyrate kinase [EC:2.7.2.7] (transferases), m phosphate butyryltransferase [EC:2.3.1.19] (transferases), n 2-hydroxyglutarate dehydrogenase [EC:1.1.99.2], p glutaconate CoA-transferase, subunit A [EC:2.8.3.12] (transferases), q unidentified, r glutaconyl-CoA decarboxylase subunit alpha [EC:7.2.4.5], s 3-hydroxybutyryl-CoA dehydratase [EC:4.2.1.55] (lyases), t 3-hydroxybutyrate dehydrogenase [EC:1.1.1.30] (oxidoreductases), u hydroxybutyrate-dimer hydrolase [EC:3.1.1.22] (hydrolases), and v poly(3-hydroxybutyrate) depolymerase [EC:3.1.1.75] (hydrolases). List of few predicted gene in these pathways: a unidentified, b pf1D or it could be pflB [50] gene , c atoBgene, and d phbB gene. However, others were designated as phaA [51–53], orphbA [54, 55] gene, d its phaB [56, 57] or phbB [58] gene, e predicted as phaC or phbB gene , f unidentified in pathway, g it could be yugJ [59], h unidentified in the KEGG-KASS pathway, i gene encoding isologs of butyryl-CoA dehydrogenase enzyme are unidentified. It could be Swol_1933 [60], k croR gene, l unidentified in pathway, n unidentified gene, p it could be gctA , r it could be gcdA (s) croR gene, t it could be Bdh2 [61] gene, u unidentified, and v phaZ gene. These metabolic pathway and respective KEEG number database were collected from KEGG [62]

From KEGG-KASS metabolic pathway (Fig. 8), it was predicted that glucose converted into pyruvate. Pyruvate then converted into acetyl-CoA by *formate C-acetyltransferase* enzyme and gene involved in encoding *formate C-acetyltransferase* has been identified as *pf1D* gene in this pathway. As displayed in Fig. 9*, acetyl*-CoA was metabolically converted into *acetoacetyl-CoA* in *B. cereus* BNPI-92 due to *acetyl-CoA C-acetyltransferase* encoded by *atoB* gene.

As illustrated in Fig. 9, *acetyl-CoA* has been metabolically converted into *acetoacetyl-CoA* by 3-*ketoacyl-CoA thiolase* or acetyl*-CoA acyltransferase* [EC:2.3.1.16] enzyme that was encoded by *fadA* gene (1173 nts, 391 aa residue) (Fig. 9) when it was performed by KEGG-KASS and RAST servers. As displayed in Fig. 9, *acetoacetyl-Co-A* was converted into *(R)-3-hydroxybutanoyl*-*CoA* by *acetoacetyl-CoA reductase* in *B. cereus* BNPI-92 strain. In this metabolic pathway, gene encoding acetoacetyl-CoA reductase was predicted as *phbB* (Fig. 9). A PHA-producing *B. cereus* BNPI-92 strain collected from an area of plastic waste harbored polyhydroxyalkanoate synthase enzymes (Fig. 9. As observed in Fig. 9, *polyhydroxyalkanoate synthase* enzyme is used to transform (R)-3-hydroxybutanoyl-CoA into poly-β-hydroxybutyrate (PHB). As predicted in the KEGG-KASS metabolic pathway, *polyhydroxyalkanoate synthase* was encoded by the *phbC* or *phaC* gene.

As displayed in Fig. 9, *butanol dehydrogenase* (1164 nts, 388aa) activated conversion of 1-butanol to butanal for the PHB biosynthesis process. The gene encoding for NADH-dependent-*butanol dehydrogenase* enzyme was unidentified in the metabolic pathway for *B. cereus* BNPI-92 strain. A *4-hydroxybutyryl-CoA dehydratase or vinylacetyl-CoA-delta-isomerase* enzymes was observed as a conversion tools of Crotonoyl-CoA to (R)-3-Hydroxybutanoyl-CoA in B. *cereus* BNPI-92 strain screened for PHA biosynthesis. In the *B. cereus* BNPI-92 strain of the PHA metabolic cycle, a short pathway was similarly observed (Fig. 9). In this pathway, acetoacetate converted into (R)-3-hydrooxybuyrate by *D-beta-hydroxybutyrate dehydrogenase* enzyme (EC 1.1.1.30).(R)-3-hydrooxybuyrate was metabolized into (R)-3-((R)-3 hydroxybutnoyloxy) butanoate by enzyme hydroxybutyrate-dimer hydrolase [EC:3.1.1.22] that involved in PHA metabolisms (Fig. 9). But, the gene involved in encoding hydroxybutyrate-dimer hydrolase was not identified. Poly (3-hydroxybutyrate) depolymerase gene was also detected in the genome sequence of BNPI-92 strain (Fig. 9).

As was illustrated in Fig. 9, 2-hydroxyglutarate dehydrogenase was used to convert five carbon compound (2-oxoglutarate) into a hydrogenated 2-hydroxyglutarate compound in *B. cereus* BNPI-92 strain metabolic pathway. In KEGG-KASS server, a gene encoding for 2-hydroxyglutarate *dehydrogenase* was identified (Fig. 9). From KEGG-KASS server, it was observed that 2-hydroxyglutarate was metabolically converted into 2-hydroxyglutaryl-CoA due to *glutaconate CoA-transferase*, subunit A [EC:2.8.3.12] that belonged to class transferase*.*

As illustrated in Fig. 9, glutaconyl-CoA may be altered into *crotonyl*-*CoA by* glutaconyl-CoA decarboxylase in the 2-oxoglutarate pathway for *B. cereus* BNPI-92 strain. Hydroxybutyrate-dimer hydrolase was identified in the genome sequence of BNPI-92 strain. Finally, PHB was produced from (R)-3-((R)-3-hydroxybutnoyloxy) butanoate when poly (3-hydroxybutyrate) depolymerase had been activated for the reaction to start in acetoacetate pathway for this hydrolases class of enzyme.

## Ribosomal multilocus sequence typing (rMLST)

In rMLST, various housekeeping genes were obtained for *Bacillus* sp. BNPI-92. These housekeeping genes include *rpsA, rpsB, rpsC*, rpsD, *rpsE, rpsF,* and others with their corresponding allele, contigs, start position, end position, and linked data values for typification of the recent our isolate (Additional File 1: Table S4). There are 20, 22, and 12 encoding the bacterial ribosome protein subunits for *rps*, *rpl*, and *rpm* genes that were predicted for *Bacillus* sp. BNPI-92 strain, respectively (Additional File 1: Table S4). As indicated in Additional File 1: Table S4, the rMLST report were found to be 100% allelic support for *B. cereus*. It was also predicted that 55 out of 55 exactly matched *B. cereus*. Other related bacterial species such as *B. albus, B. bombysepticus, B. paranthracis, B. thuringiensis, B. tropicus,* and others (Additional File 1: Table S4).

## Discussion

A range of k-mers from 31 to 95 was used for Velvet and ABySS assembly. Our result demonstrated that Velvet assembly was used with kmer 47 for all further downstream analysis. It was a better statistics than all other assembly generated for the complete assembly statistics made using QUAST 4.0 and BUSCO 2.0 for bacterial genome draft. In contrast to this study, ABySS and IDBA-UD assembling tools showed a good performance for the fungal genomic draft in terms of memory, running time, and quality [63]. In our result, a considerable amount of contigs and genomic length was predicted. In line with this study, it was reported that [64] all obtained contigs by Velvet software that used to generate scaffolds with average size of 108,565 bp in which the minimum and maximum scaffold sizes of 144 and 1,059,836 bp [65], respectively, are lower than the current of our results. Abdelhafiz and his co-author found that a total of 34 gaps from 41 gaps were closed. In addition, after gap closing, they found a total of 39 scaffolds with an average size of 108,580 bp [65]. Velvet assembler generated GC contents and N_50_ for *Bacillus* sp. BNPI-92 which is a related GC content reported by Lin et al. (2011) using Velvet and other tools. However, compared to the present prediction, a higher number of uncalled bases (gaps) (N_50_, 488,188) [66] was detected using the same tools for *B. subtilis* Strain QB928.

It was realized that the RAST server was able to identify a set of gene calls with their respective function, location, and level of protein expression. A GC content was similarly reported for *Bacillus* sp. BNPI-92 strain which is a similar percentage reported for *B. cereus* (35.14–35.38%) that is isolated from Foodstuff [67]. In agreement with this study, SEED subsystem categories [68] and the functional gene for *B. cereus* FORC_013 had been identified. It was also found that *B. cereus* FORC_013 have a genomic feature (5,418,913 bp WGS and 35.3% GC contents) similar to our strains. It was suggested that our strain may share the species level with *B. cereus* FORC_013. A single circular plasmid was similarly detected in FORC_013 WGS which is a conflicting finding to our prediction for *Bacillus* sp.BNPI-92 strain screened for PHA biosynthesis.

Subsystem category distribution (pie-chart) and subsystem feature counts were also predicted after BNPI-92 genomic DNA annotation had been performed using the RAST server. These subsystems are supposed to be used for cellular function and metabolisms. Strengthening our suggestion, it was predicted that subsystems used to provide general information about metabolism, to improve quality of annotation, and offer a framework for establishing the statistical properties needed to effectively exploit these tendencies [69]. Carbohydrates and other organic compounds were predicted in our result. It was suggested that these organic compounds might have been used to initiate PHA biosynthesis. *Acetyl-CoA thiolase, acetoacetyl-CoA reductase* and *polyhydroxyalkanoic acid synthase* were similarly predicted for PHA biosynthesis process in BNPI-92 strain using RAST server which supposed to use for PHA accumulations.

In this result, *Acetyl-CoA thiolase, acetoacetyl-CoA reductase* and *polyhydroxyalkanoic acid synthase* were predicted for BNPI-92 strain which is a similar genome annotation reported for *Pseudomonas extremaustralis.* These genes could have been employed for PHB production and other metabolic processes [70]. In *P. extremaustralis*, coding sequences (CDS) (5934) and structural RNAs (49 tRNAs) (62) [70] had been predicted from its annotated WGS, a lower tRNA than RNA predicted for BNPI-92 strain. This indicates that both strains may share only less sequence in common.

Biochemically, it was checked that *Bacillus* sp. BNPI-92 is a gram-positive, and endospore former using RAST server, these features were confirmed for BNPI-92 strain as depicted in expandable down in cell wall and capsule (180) and dormancy and sporulation (149) subsystem feature counts. Similarly, these biochemical tests were reported for *B. cereus* [67] obtained from food stuff and *B. cereus* FORC_005 strain [71], a food-borne pathogen isolated from the soy sauce braised fish cake with quail egg.

In the present study, genomic features had been predicted for *Bacillus* sp. BNPI-92. In line with this study, it was stated that GC content (35.6%. 35.11%), circular chromosome (5,221,581 bp, 4.82 Mb), protein-coding genes (5,415, 5132), rRNA (14, 2), and tRNAs (104, 21) had been reported for *B. cereus* ATCC 14579^T^ and *B. cereus* 25, respectively [72, 73]. It is a similar feature of *Bacillus* sp. BNPI-92, a recently isolated strain for PHA biosynthesis [74]. It has been suggested that *Bacillus* sp. BNPI-92 strain could have belonged to them at the species level. Similarly, related GC content (35.0%), circular chromosome sequence size (5,218,947 bp), ORFs (5480), protein-encoding genes (5309), tRNA (96), and rRNA (10) were predicted for *B. anthracis* H9401 strain collected from Korean Patient with Anthrax [75]. Despite being essentially similar in terms of genetic feature, the current strain is not pathogenic rather it was found to be economically important and industrially applicable.

Genomic comparison for *Bacillus* sp. BNPI-92 and these five closely related bacterial strains were displayed in the occurrence table and Venn diagram graphics based on their protein sequences. In line with this study, comparative genomic analyses were performed between *B*. *cereus* 905 and other three *B. cereus* (*B*. *cereus* AR156 (CP015589), *B*. *cereus* LCR12 (MCAX00000000), and *B*. *cereus* UW85 (LYVD00000000)) using Ortho Venn^2^ for their protein sequences. It was found that these four strains shared a large set of core genome (63.9% to 72.8%) with a similar protein-coding genes sequence [76]. In agreement with this study, a similar analysis was performed for different *Streptomyces* species and these species contained a common orthologous gene cluster [18]. Genome comparative analysis had been similarly performed for eight species of *Streptomyces* involved in genome plasticity and common orthologous cluster gene groups were found [77] using the same tool.

Gene clusters were shared between *Bacillus* sp. BNPI-92 and other related strains (2930 gene clusters). This indicates that *Bacillus* sp. BNPI-92 strain shared a common protein sequence with these six aligned and closely related *Bacillus* strains. It was similarly reported that common gene clusters (2501 cluster count) had been predicted between *Streptomyces* species [18]. These common genes clusters may be suggested to be a core genome. This suggests that these strains are homologous to one another and may arise from a common ancestor. Additionally, this conserved region for these all strains might be suggested by their gene conservation in the lineage after speciation and confirms the similarity between these strains due to common protein sequence.

In Venn diagram results, a similar gene clusters (2930) were shared among these related strains, a comparable work reported for *Streptomyces* species with 2501 cluster count [18]. A number of orthologous protein-coding gene clusters shared by these strains were also represented in Venn diagram graphics. These cluster genes are common to all these strains. Probably, it could be a core genome belonging to these strains. This suggests that these strains are homologous to one another and may arise from a common ancestor. Additionally, this conserved region for these all strains might be suggested by their gene conservation in the lineage after speciation and confirms the similarity between these strains due to common protein sequence.

The common gene cluster for these strains (core genome orthologs) is fewer than the number reported for eight *Streptomyces* (2501) [18] and six *Streptomyces* species (3096) [77] genomes characterized for genome plasticity. In line with this study, several of common orthologous protein-coding gene clusters (3998) were also reported among four *B. cereus* strains which are a higher core genome (2930 gene clusters) reported for *Bacillus* sp. BNPI-92.

It was demonstrated that more overlapping gene clusters had been detected between BNPI-92 and *B. cereus* ATCC 14570^T^ and it was suggested that this strain is more closely related to *B. cereus* ATCC 14570^T^ (AE016877). The phylogenetic tree for BNPI-92 (OP329213) strain was also close allied with *B. cereus* ATCC 14570^T^ [74]. Non-over lapping gene clusters were similarly observed for these corresponding strains and it was suggesting that these gene clusters were unique to them. It indicates a unique number of coding sequences (CDS) which may be served as fingerprinting. In line with this study, common orthologous protein-coding gene clusters, overlapping and non-overlapping gene clusters were predicted for *B. cereus* 905 and other *B. cereus* strains [76].

Pair heatmap was used for genome comparative analysis to visualize the overlapping gene clusters for these strains in a pairwise fashion. Overlapping gene clusters were happened among *Bacillus* sp. BNPI-92 and these strains. It was suggested that *Bacillus* sp.BNPI-92 shared more sequence similarity with *B. cereus* ATCC 14579^T^. From RAST server, it was similarly confirmed that *Bacillus* sp. BNPI-92 strain could be *B. cereus* ATCC 14579^T^ type strain. In line with this study, overlapping gene clusters were performed for few *Streptomyces species*. The maximum and minimum thresholds of overlapping clusters were also reported for *Streptomyces albidoflavus*, *S. cattleya*, *S. fulvissimus*, *S. globisporus, S. lividans*, *S. rapamycinicus*, *S. sp.Mg1*, and *S. sp. Sv* ACTE SirexAA [18]*.* The more the shared number of gene cluster among strains, the higher overlapping gene cluster which suggested that these organisms have more common gene cluster as it witnessed. Similarly, *Staphylococcus caeli* and *S. xylosus* were also found to be closely related with each other [78]. The same authors further confirmed that these isolates were shared clade with *S. hemolyticus*. But, in our previous publication for *Bacillus* sp., BNPI-92 [74] was found to share clade with *B. cereus* ATCC 14579^T^ type strain as indicated in the phylogenetic tree for 16S rRNA gene sequences. Based on 16S rRNA gene sequencing, it was confirmed that *Lactobacillus paracasei* PCR140 shown 97% similarity *Lacticaseibacillus paracasei* which are probiotic bacterial species [79]. The *L. fermentum* NMCC-14 [80] showed sequence similarity with other species lactic acid bacterial species.

This core genome is predicted to have Gene Ontology (GO) that is subdivided into biological processes, molecular functions, and cellular components based on functional information associated with different gene clusters or gene families, a similar report by [81]. BNPI-92 strains have certain functionally annotated and a secondary metabolite that is able to encode orthologous gene clusters associated with biological and cellular metabolisms. In line with study, it was similarly reported that the biosynthetic gene clusters [77] were able to produce secondary metabolites in Streptomyces. It was also recorded that secondary metabolism encoding gene clusters (cluster 67, 66, 13, 12, and 7) [77] were predicted for five Streptomyces genomes.

Gene clusters associated with sporulation resulting in the formation of a cellular spore was predicted in core genomics of these isolates and suggested to be used for resistance to harsh environmental conditions. It also confirms our biochemical test for *Bacillus* sp. BNPI-92 strain screened PHA biosynthesis. In the core genome, it was similarly observed that a gene family is associated with butyrate metabolic process and it was suggested that this gene cluster could have been used to encode protein involved in PHA biosynthesis. In line with this suggestion, a P (3HB-4HB-3HV) terpolymer had been produced from a metabolic process of butyrate, valerate, and 4HB [54]. It was added that the polymerization of 3OH-butyrate monomers was assisted with PHB synthase that encoded *phaC* gene [82, 83]. *Bacillus* sp. INT005 which is the same genus to our isolate is able to metabolize butyrate to produce PHB, strengthening results to our estimation [84].

The prediction of 3-hydroxybutyrate dehydrogenase GO in this result could have been associated with PHA degradation. This is probably a reason for decreasing PHA production after 72 h onward as indicated in our previous study for the same PHA-producing isolate [74]. Similarly, it was reported that gene encoding 3-hydroxybutyrate dehydrogenase (Hbd) that can degrade PHA was detected in *Paracoccus denitrificans* PD01 [85]. Gene ontology for β ketothiolase (GO:0003988), acetoacetyl-CoA reductase (GO:0018454), and polyhydroxyalkanoates synthase (GO:190,144) activity have been founded in PHA-producing microorganisms [83, 86].

The biological process GO prediction in terms of carbohydrates (GO: 0005975) and metabolic process (GO: 0008152) strongly estimated to be used for PHA biosynthesis in the BNPI-92 strain (data not shown). Similarly, the presence of transcription DNA template (GO: 0006351) and regulation of transcription DNA template (GO: 0006355) may be used for cellular structure and function. In line with this study, the most conserved functional groups of GO of biological process such as cation transport (GO: 0006812), ion transport (GO:0006811), and single-organism transport (GO:0044765) [87] were reported for *B. cereus.*

The presence of ATP binding, catalytic activity, and DNA binding GO in the BNPI-92 strain was confirmed as a molecular function in a comparable report for *B. cereus* (626 number of terms) [87]. But, it is a higher molecular function GO term than the current GO term reported for our strain. It was also indicated that our strain might have shared sequence similarity with *B. cereus* [87]*.* The catalytic activity was similarly confirmed from subsystem information of RAST server genome annotation a similar report for *B. cereus* ATCC 14579^T^ (NC_004722) strain [88] for certain extracellular enzymatic activity such as amylase, catalase, and protease.

Cellular component category is among milestones for GO such as integral component of membrane, cellular component GO term, and cytoplasm GO term for identification of specific gene function in this strain. In line with this study, membrane-associated category GO was reported as a cellular component in *Bacillus B. cereus* that is associated with specific disease manifestations [87]. For *B. subtilis* essential genes, it was found that the cellular component GO terms with the highest enrichment score were predicted for cytoplasm (GO:0005737) and ribosome (GO:0006412) [89]. This suggested that the recent BNPI-92 strain did not share essential core genes with *B. subtilis* in terms of ribosome GO. However, *B. subtilis* and the BNPI-92 strain have certain common essential GO terms like cytoplasm GO (GO:0005737) category terms.

Genome comparative analysis between these closely related strains can be achieved by using OrthoANI, Origin Ani, and ANI calculator. The OrthoANI obtained between BNPI-92 and *B. cereus* ATCC 14579^T^ strain (98.88%) genomes was higher than OrthoANI values reported between strain GFP-2 and *B. siamensis* KCTC 13613 T (94.1%) and GFP-2 strain and *B. amyloliquefaciens* DSM 7 T (94.4%) [90]. It was similarly reported that *B. licheniformis* CBA7126 strain was closest to *B. licheniformis* VTM3R78 (99.99% orthoANI), followed by *B. licheniformis* B4164 (99.98%), *B.* sp. H15-1 (99.85%), *B. licheniformis* B4124 (99.81%), and *B. licheniformis* V30 (99.80%) [91] which are generally higher sequence similarity reported for *Bacillus* sp. BNPI-92 strain.

Origin ANI was also evaluated for comparative genome analysis for BNPI-92 and other closely related strains. Considering the finding of [17] benchmark for ANI (> 95–96%) and demarcation range set [31] for certain strains, it was indicated that the two strains belong to *B. cereus*. Therefore, the recent PHA-producing *Bacillus* sp. BNPI-92 strain isolated from landfill was confirmed to be a *B. cereus* type strain. It was delineated to *B. cereus* ATCC 14579^T^ type strain. Besides, based on analysis of original ANI value reported for CBA7126 genome sequences (with the symmetric identity of > 97%) [91] (> 99%) and its conclusion, *Bacillus* sp. BNPI-92 strains that showed 98.25% genome sequence similarity with *B. cereus* ATCC 14579^T^ suggested as *B. cereus* type strain.

An *is*DDH was used to estimate the relatedness among BNPI-92 and the rest strains. It was found that 73.73% sequence similarity had been detected between BNPI-92 and *B. cereus* ATCC 14579^T^ strain. This indicated that BNPI-92 was suggested to be a *B. cereus* species based on a benchmark set for *is*DDH [40]. Thus, the BNPI-92 strain was delineated to a *B. cereus* species. In line with this study, about 43 *B. cereus* groups (BCG) were characterized with *is*DDH and found that cluster BCG03 yielded *is*DDH values ≥ 70%. Similarly, *is*DDH was performed for 15 strains delineation and found that *is*DDH values were ≥ 70% for cluster BCG04 that proposed to be *B. thuringiensis* type strain of [40]*.* In agreement with this study, *is*DDH values were predicted between *Aeromonas veronii* and the other two strains were slightly below 70% [40]. Except for *B. cereus* ATTCC 14579^T^ strains, the remaining strains shared a ≤ 70% genome sequence with BNPI-92 strains. Thus, based on [31] finding, BNPI-92 strain did not share sequence similarity to a species level with these strains. Strongly, it was confirmed that in *is*DDH values, ≥ 70% genome sequence similarity between two species was recommended as a cut-off point for the same species delineation [31].

Comparative genome analysis similarity among strains were checked by ANI calculator to estimate their genome sequence similarity using best hits (one-way ANI) and reciprocal best hits (two-way ANI) as per calculated by [31]. The ANI calculator used to estimate the ANI using best hits (one-way ANI) and reciprocal best hits (two-way ANI) between two genomics which were 99.04% (from 3277) and 99.10% (from 3206) fragments, respectively, as founded by Goris et al. [31]. It strongly confirms BNPI-92 strain to be *B. cereus* strain type based on Goris et al.'s [31] suggestion. A predicted, ANI between BNPI-92 and *B. wiedmannii* FSL W8-0169 strains which is 90.79% suggests that the BNPI-92 strains were different from *B. wiedmannii* FSL W8-0169 strains at both species and strain levels and it should not be named as *B. wiedmannii*. In line with this study, ANI was performed for strain AR156 and other *B. cereus* strains (905, UW85, and LCR12) [76]. It was found that their sequence similarity by ANI is lower than 93% and it was suggested that strain AR156 may represent a species other than *B*. *cereus.* The prediction of maximum ANI between BNPI-92 and *B. cereus* ATCC 14579^T^ strain (99.1%) strongly confirms that BNPI-92 strain should be named as *B. cereus* BNPI-92 strain.

In conclusion, based on a combination of RAST annotation of genomic DNA, OrthoVenn tool, *in silicon* DDH (*is*DDH), OriginalANI, and OrthoANI results, *Bacillus* sp.BNPI-92 strains were confirmed to be a *B. cereus* type strain*.* Similarly, [91] comparative genomic analysis was performed for *B. licheniformis* CBA7126 using a certain related sequence of *B. licheniformis* strains.

The TYGS result indicated that BNPI-92 strain has been identical with *B. cereus* ATCC 14579 strain at species and subspecies. The result for Delta value suggests that the constructed tree-likeness for our current phylogenetic tree was accurate as it was stated by [92]. The same authors stated that when δ approaches zero, the distance matrix for phylogeny is found to be accurate. For instance, if the δ value of the resulting distance matrix is 0.1629, globally, the highest distance quality obtained [92] is higher than the value obtained. However, it was revealed that operational taxonomic units (OTUs) with high δ values are able to provide a poor phylogenetic tree or less tree-like data. It could be due to sequence contamination or sequence incompleteness.

It was found that vitamin, sugar metabolism, and genes involved in fructose/mannose, amino/nucleotide sugar metabolisms were expressed at the highest level in date palm (*Phoenix dactylifera,* L.) at some stages of growth [93]. Glycolysis or gluconeogenesis and vitamin B6 metabolisms were similarly expressed in BNPI-92 and *B. cereus* ATCC 14579^T^ strain which were similarly detected in date palm tree [93]. This result suggested that these strains and date palm could have conserved genes for glycolysis and vitamin production though their classification in terms of kingdom or domains were distinct. It was detected that *Bacillus* sp.BNPI-9 shared relatively a similar level of gene expression for zeatin biosynthesis, vitamin B6 metabolisms, valine, leucine and isoleucine degradation, valine, leucine and isoleucine biosynthesis, and polyketide sugar unit biosynthesis metabolic pathways with *B. cereus* ATCC 14579^T^ for a given metabolic pathways. This could be used to estimate their evolutionary relationship based on a given gene encoded KEGG metabolic pathway.

BNPI-92 strain used to produce PHA biosynthesis encoding genes (*phaA, phaB and phaC*) that suggested it was used for PHA polymerization. These genes were predicted when the De Novo assembly whole genome sequence had been computed and annotated in the RAST server system. In line with this study, it was found that genes such as *phaA* (1179 nts), *phaB* (738 nts), and *phaC* (1767 nts) that able to encode PHB biosynthetic enzyme were predicted for *Ralstonia eutropha.* These genes were organized on the same operon and designated as *phaCAB* [94]. In line with this study, the *phaC* gene sequence was reported in different *Bacillus* species genomic DNA such as *B. megaterium, B. cereus*, and *B. anthracic*. This confirms the current result for *phaC* gene predicted in annotated genomic DNA of BNPI-92 strain in Rast server or subsystem. The PHA polymerases or PHA synthases are able to catalyze 3-R-hydroxyalkyl CoA thioesters into PHA [95–98].

RAST server analysis showed that *acetoacetyl-CoA reductase* is able to metabolize and convert a*cetyl-CoA* to butyrate in PHA biosynthesis pathways. It was also confirmed that *acetoacetyl-CoAreductase* encoded by *phaB* gene is able to form PHB. The *acetoacetyl-CoA* reductase protein-coding gene (*phaB*) was reported in *R. eutropha* H16 genome sequence and harbors isologs *phaB*^*2*^, *phaB*^*3*^*,* and *phaB*^*15*^ [54, 99]. In line with this finding, an isologous isologs *phaA* gene sequences were similarly detected in annotated BNPI-92 strains and designed as *phaA*^*1*^, *phaA*^*2*^*,* and *phaA*^*3*^. They encode *β-ketoacyl-CoA thiolase* or *acetyl-CoA acetyltransferase.* In agreement with this assumption, *phaA is stated* as an encoding gene for *β-ketoacyl-CoA thiolase* or *acetyl-CoA acetyltransferase* enzyme and plays a role in PHB formation [54]. These enzymes were predicted in RAST server pipeline and suggested for the conversion of acetyl-CoA to butyrate and promote polyhydroxybutyrate metabolic processes.

The existence of *PhaR* gene sequence BNPI-92 strain genomes suggested that this gene may be used to suppress PHA biosynthesis protein such as *phaP* [100], a well-known enzyme reported for encoding phasin protein and PHA granules polymerization along with *phaC* gene [95]. The prediction of this gene strongly confirms time course PHA biosynthesis of our result in which the PHA biosynthesis (data not shown) and Nile Blue A staining (data not shown) were decreased after 72 h onward. It was presumed that the *phaR* repressor protein may be over-expressed as the incubation period goes on. In contrary to this suggestion, a gram-positive poly (3-hydroxybutyrate) (PHB)-degrading *B. megaterium* N-18–25-9 harbors PHB depolymerase gene designed as *PhaZ* (Bm) [101] which is a confirming finding with our results in KEGG-KASS metabolic pathways.

Gene suggested to encode *3-hydroxybutyryl-CoA dehydratase* enzyme (*croR* gene) was predicted in these strain and suggested that these strains have a common gene sequence that used to express this protein. After genome annotation had been performed, it was realized that *3-hydroxybutyryl-CoA dehydratase* enzyme plays an essential role for acetyl-CoA fermentation to butyrate and PHB metabolism*.* In line with this study, it was found that *3-hydroxybutyryl-CoA dehydratase* enzyme was able to hydrate crotonyl-CoA to 3-hydroxybutyryl-CoA and lead to PHB biosynthesis in the metabolic pathways [102]. However, in *Clostridium aminobutyricum* metabolic pathway, *3-hydroxybutyryl-CoA dehydratase was* used to hydrate crotonyl-CoA to 3-hydroxybutyryl-CoA and oxidized to acetoacetyl-CoA, which is finally cleaved to two acetyl-CoA (Buckel, 2010).

The BNPI-92 contained *pha* gene loci such as *phaP, phaQ, phaR, phaB*, and *phaC* on the same operon and designated as *phaPQRBC* gene cluster. The *phaP* and *phaQ* genes were located in the opposite direction to *phaR*, *phaB*, and *phaC* which is boldly agreed with PHA genetic organization for *B. megaterium* subgroup [98]. It was predicted that the *phaQ* gene encodes a P(3HB)-transcriptional regulator (*phaP*) protein as predicted in the RAST server. Similarly, it was stated that *phaQ* gene is a PHB-responsive repressor due to *phaP* expression levels being blocked [103]. However, in *B. megaterium* with *phaPQRBC* gene cluster, PHA can be synthesized when it had been transferred to *E. coli, Pseudomonas putida*, and *B. subtilis* [95, 104], a similar result with our suggestion of its role for PHA biosynthesis in the present study.

However, in KEGG-KASS server, a gene encoding for *enoyl-CoA hydratase* and *3-hydroxyacyl-CoA dehydrogenase* were separately predicted as *fadB* and *fadN,* respectively*.* However, in contrast to this perdition, [5] designed R-specific *enoyl-CoA hydratase* encoding gene as *phaJ*. Gene encoding for *acetoacetyl-CoA synthetase* was proposed as *acsA* based on [49] report, and similarly, it was predicted in BNPI-92 strain. Poly-3-hydroxybutyrate degradation affecting pathway had been identified in *Sinorhizobium meliloti* chromosomal loci as acetoacetyl coenzyme A (acetoacetyl-CoA) synthetase (encode by *acsA* gene) with 72,000 kDa molecular weight. These findings suggest that acetoacetyl-CoA synthetase tends to activate acetoacetate to acetoacetyl-CoA in the *S. meliloti* for poly-3-hydroxybutyrate accumulation [49].

We realized that the presence of *phaA, phaB*, and *phaC* genes in our BNPI-92 strains suggested that this strain has a capability to synthesize short chain length (*sch*PHA) biopolymer. In the present study, FTIR, XRD, and NMR characterization for the same strain similarly confirm this assumption (data not shown). In line with this study, it was confirmed that *Bacillus* strains such as *B. megaterium* and *B. cereus* harboreda PHA biosynthesis gene [98] designated as *phaC* despite being essential unlike in nucleotide sequence. The same authors stated that these species tend to produce short-chain-length monomers such as 3-hydroxybutyrate (C_4_) and 3-hydroxyvalerate (C_5_) for PHA polymerization [98] which is strong evidence for our current suggestion. It was stated that *phaB* used to encode an NADPH-dependent *acetoacetyl-CoA reductase* (*PhaB*) that plays a vital role for the conversion of (R)-3HB-CoA monomer to PHA polymerization whereas *phaR* and *phaC* genes encode PHA synthase subunits [103] which is a similar finding with our current suggestion for a *phaPQRBC* gene cluster roles.

A genomic feature of the *phaP* gene predicted in BNPI-92 strains was *not yet* part of a subsystem in the RAST server pipeline. This gene may not be expressed despite existing on the same operon along with *phaB, phaC*, and *phaR* genes. It was reported that the *phaP* gene was used to encode PHA granule-associated protein (*PhaP*) [103] which is a non-enzymatic protein (Phasin) that is located on the surface of PHA granules within the microbial cell membrane. It was reported to function for blocking unnecessary proteins binding to PHA granules [103].

Once the metabolic pathway is well-known, it is more essential to produce large amounts of polyhydroxyalkanoate (PHA) polymers from microorganisms using a recommended carbon source. We realized that glucose conversion to pyruvate was activated by *formate C-acetyltransferase* enzyme that was encoded by *pf1D* gene in BNPI-92 strain. However, in *L. sakei* carbohydrate conversion, pyruvate was activated by *formate C-acetyltransferase (pyruvate formate-lyase) (formate acetyltransferase*) [50] that encoded and identified as *pflB* gene.

An *atoB*gene was used to encode *acetyl-CoA C-acetyltransferase* that is able to metabolically convert *acetyle*-CoA into *acetoacetyl-CoA* in the BNPI-92 strain. Similarly, in metabolic engineering of *E.coli, acetyl-CoA C-acetyltransferase* (encoded by *atoB* gene) [105] engaged in *1,3-Butanediol* biosynthesis process, a strongly supporting report for our prediction, despite distinct strain involved in the metabolic process. This strain could have a common *atoB* gene that is essentially involved in metabolisms and carbon source utilization. Biosynthesis of 1,3-butanediol was observed when acetyl-CoA C-acetyltransferase [105] had been overexpressed by metabolically engineered *E. coli*. The same authors further stated that *acetyl-CoA C-acetyltransferase* can be under-expressed due to aldehyde dehydrogenase activity in spite of *atoB* gene overexpression [105].

It was shown that *acetyl-CoA* has been metabolically activated into *acetoacetyl-CoA* by 3-ketoacyl-CoA thiolase or acetyl*-CoA acyltransferase* (encoded by *fadA*) enzyme. In consistency with this study, it was found that two *acetyl-CoA* molecules had been condensed to acetoacetyl-CoA in the form of *Zoogloea ramigera* and *Halomonas boliviensis* [51, 52, 106]. However, the enzyme involved in the conversion of acetyl-CoA to acetoacetyl-CoA was identified as 3-ketothiolase. Its gene was similarly designated as *phbA* [54]. Similarly, it was stated that β-ketoacyl-CoA thiolase (encoded by *phbA* gene) was able to condense two molecules of acetyl-CoA into acetoacetyl-CoA in *fluorescent Pseudomonads* [55]. Davis et al. [56] identified a new pathway b-ketothiolase and NADPH-dependent acetoacetyl CoA reductase pathway that encoded *phaA* and *phaB* gene *Bacillus* sp. 256 isolated from soil samples. Five genes namely *phaA, phaB, phaR*, *phaC,* and *phaP* were similarly reported for *B. cereus* strain tsu1 strain [57] which is a similar isolate at species with our newly isolated strain.

Acetoacetyl-CoA reductase was used to convert *acetoacetyl-Co-A* into *(R)-3-hydroxybutanoyl*-*CoA* in *B. cereus* BNPI-92 strain. In line with this study, in PHA biosynthesis pathway, *phbB* was found to be used for encoding NADPH-dependent acetoacetyl-CoA reductase enzyme that tends to convert acetoacetyl-CoA to 3-*hydroxybutyryl*-*CoA* [54] in *Z. ramigera*. However, from the same pathway, *phaB* isologous genes, namely *phaB*^*2*^, *phaB*^*3*^*,* and 15 others were identified in *R. eutropha* H16 [58]. The gene sequence analyses showed them as paralogs originated from gene duplication events [58]. It was further confirmed that NADPH-dependent acetoacetyl-CoA reductase (encoded by *phbB*) was able to reduce acetoacetyl-CoA to (R)-3-hydroxybutyryl-CoA [55] in *F. pseudomonads* metabolic pathway.

In the genomic sequence of the BNPI-92 strain, *phaC is predicted* to be used for PHA biosynthesis, similar information with the review paper written by [107]. P(3HB) polymerase engaged in (R)-3-hydroxybutyryl-CoA monomers polymerization into short-chain length PHB. It was encoded by *phbC* gene [55] in *F. pseudomonads,* which strongly confirms our prediction. Despite similar genes reported from different genera, these bacterial genera may share certain common gene sequence in evolutionary history. PHA biosynthesis due to PHA polymerases (*PhaCs* gene) had been reported for *B. megaterium* and *Rhodospirillum rubrum*. The PHA polymerization (PHB) was facilitated with PHA synthase enzymes that had been reported for the conversion of 3-R-hydroxyalkyl CoA thioesters to PHAs [96, 108, 109].

B*utyryl-CoA dehydrogenase* encoding gene was detected in the genome of *Syntrophomonas wolfei subsp. wolfei* (strain DSM 2245B), similar result predicted in BNPI-92 strain. It was a slow growing anaerobic and evolutionarily adapted bacteria for syntrophic growth with methanogens and other hydrogen or formate using microorganisms in the natural ecosystem [60]. Its gene was identified as Swol_1933 [60].

A 3-hydroxybutyrate dehydrogenase-encoding gene (*bdhA*) had been cloned and sequenced from *Rhizobium (Sinorhizobium) meliloti* [61]. It was reported that the gene contains 777 bp open reading frame that encodes a polypeptide of 258 amino acid residues, strongly similar to our prediction for gene size. *bdhA* is the first gene transcribed in an operon that also includes *xdhA*, encoding xanthine oxidase/dehydrogenase [61] in *R. meliloti*.

It was found that 2-hydroxyglutarate dehydrogenase is involved for PHA biosynthesis in the BNPI-92 strain. However, in *Clostridium acetobutylicum, Corynebacterium glutamicum, Escherichia coli,* and *R. eutropha* transgenic cell*,* 2-hydroxyglutarate dehydrogenase overexpression had been reported for crotonic acid production from 2-oxoglutarate [110]. This could be due to different strain types involved in the metabolic process. It was stated that 2-hydroxyglutarate dehydrogenase had been encoded by *lhgD* in *E. coli* k12 which is a contradictory result to our prediction. Similarly, it was reported that gene encoding for *glutaconate CoA-transferase*, subunit A was detected in *Acidaminococcus fermentans (strain ATCC 25085 / DSM 20731 / VR4) and named as gctA* [111]*.*

Although mechanisms of action were unknown, in this pathway, glutaconyl-CoA decarboxylase was suggested for PHA biosynthesis. However, 2-hydroxyglutarate dehydrogenase is reported for crotonic acid production [112], which has an additional role in 2-oxoglutarate pathway. In agreement with our estimation, it was stated that conversion of glutaconyl-CoA to crotonyl-CoA was activated by glutaconyl-CoA decarboxylase and glutaryl-CoA dehydrogenase in *Pseudomonas*, *Rhodomicrobium annielii, R. palustris,* and *Rhodocyclus purpureus* [113]*.* Although currently not detected in *B. cereus* BNPI-92 strain, a gene encoding for glutaconyl-CoA decarboxylase had been reported as *gcdA* in *Acidaminococcus fermentans (ATCC 25085 / DSM 20731) type strain* [111].

Hydroxybutyrate-dimer hydrolase plays a vital role for hydrolysis of (R)-3-hydroxybutyrate (3HB) to (R)-3-((R)-3-hydroxybutnoyloxy) butanoate in *B. cereus* BNPI-92 strain for acetoacetate metabolic pathway. Finally, PHB was produced by poly (3-hydroxybutyrate) depolymerase when *B. cereus* BNPI-92 strain used (R)-3-((R)-3-hydroxybutnoyloxy) butanoate as a carbon source. The presence of this enzyme suggests that *phaZ*, a depolymerase enzyme, could have existed in *B. cereus* BNPI-92 strain, a similar PHA gene profile reported in *B. megaterium* [95]. In line with our estimation, it was stated that intracellular D(-)-3-hydroxybutyrate (3HB)-oligomer hydrolase gene from *R. eutropha* (*Alcaligenes eutrophus)* H16 was cloned, sequenced, and characterized as *phaZ* [114].

The rMLST is an approach that indexes variation among different housekeeping genes that encoding the bacterial ribosome protein subunits. In this study, using rMLST, genes such as *rps*, *rpl*, and *rpm* genes were anticipated for *Bacillus* sp. BNPI-92 strain. Study showed that *rps* and *rplX* genes were identified from the genomic DNA of *Streptococcus pneumoniae* [115] using rMLST which is a similar finding to the recent prediction*.* This indicated that these genes may be conserved among *Bacillus* and other bacterial species. The rMLST report had supported 100% *B. cereus*. Other related bacterial species were also predicted during rMLST analysis. They are mainly *B. albus, B. paranthracis, B. thuringiensis,* and *B. tropicus*.

## Conclusions

Whole genome sequence was performed and PHA-producing *Bacillus* sp. BNPI-92 was delineated to a specific category. In this identification, SPAdes, ABySS, and Velvet software were employed for de novo assembly, and velvet was chosen for analysis since it has better statistics. A total average of 73.85714 bp contigs was generated using Velvet assembler with an average length of 698,773 bp and with minimum and maximum length of 982,387 and 5,527,513 bp, respectively. In this WGS analysis, 5719 genes were predicted. About 5652 genes were predicted to be significant. Genome annotation was performed using the UniProt database, BLASTX program, and RAST server for *Bacillus* sp. BNPI-92 strain genome assembly to identify functional gene, gene location, and distribution. The assembled genome sequence of *Bacillus* sp. BNPI-92 strain was annotated to identify functional genes, their location, and arrangements using RAST server. It was found that the recent PHA-producing strain was related to *B. cereus* ATCC 14579 in terms of GC %, sequence length, number of coding sequence, and size and location of PHA expressing genes such as *phaA, phaB*, and *phaC* genes. Multiple genomic comparisons were performed and the recent strains and its related strains have shared 1231 gene clusters which were conserved, and probably, it could be a core genome belonging to these strains. The sequence of the recent strain was annotated using RAST server and it was found that this strain could be *B. cereus* ATCC 14579 type strains based on genome information and their gene features. In the present study, 16SrRNA gene sequence was extracted from WGS using EzBioCloud to identify the BNPI-92 strain. It was found that BNPI-92 has 100% sequence similarity with *B. cereus* ATCC 14579. Orthologous Average Nucleotide Identity (OAT) tools such as OrthoANI, Original ANI, and GGDC of 16SrRNA were performed for *Bacillus* sp. BNPI-92 comparative genomic analysis. The result showed that *Bacillus* sp. BNPPI-92 was the closest strain to *B. cereus* ATCC 14579 with 98.88% OrthoANI followed by *B. thuringiensis* (92.09%, OrthoANI), *B. wiedmannii* FSL w8-0169 (91.76%, OrthoANI), *B. paranthracis*Mn5 (91.54), and (98.89%, OrthoANI) and *B. anthracis* Ames (91.70%, OrthoANI). Based on similarity values > 95–96%, the current PHA-producing *Bacillus* sp. BNPI-92 isolated from landfill was strongly confirmed to be a *B. cereus* type strain. Finally, it was designated as *B. cereus* BNPI-92. Genetic organization was predicted for *Bacillus* sp. BNPI-92 using RAST server to determine a PHA biosynthesis encoding gene. It was found that *phaP*, *phaQ, phaR, phaA*, *phaB*, and *phaC* were found in both *B.* cereus BNPI-92. These genes were located on the same operon except for *phaA*. It was designated as *phaPQRBC*.

### Supplementary Information

**Additional file 1.**

## Data Availability

All discussed data have been included into this manuscript.

## Abbreviations

ANI: Average nucleotide identity
BNPI: Bhubaneswar Nandankanan PHA-producing isolate
GGDC: Genome–Genome Distance Calculator
*is*DDH: In silicon DNA-DNA hybridization
KEGG: Kyoto Encyclopedia of Genes and Genomes
OrthoANI: Orthologous Average Nucleotide Identity
GO: Gene Ontology
OAT: Orthologous Average Nucleotide Identity Tool
OrthoANI: Orthologous Average Nucleotide Identity
RAST: Rapid Annotation using Subsystem Technology
