# Supplementary material for: De novo assembly and comparative genome analysis for polyhydroxyalkanoates-producing Bacillus sp. BNPI-92 strain
Source: J Genet Eng Biotechnol. 2023 Nov 22;21:132. doi: 10.1186/s43141-023-00578-7 (PMC10665291; doi:10.1186/s43141-023-00578-7)
Supplement: Supplementary file 1 — Additional file 1. [file 43141_2023_578_MOESM1_ESM.doc]

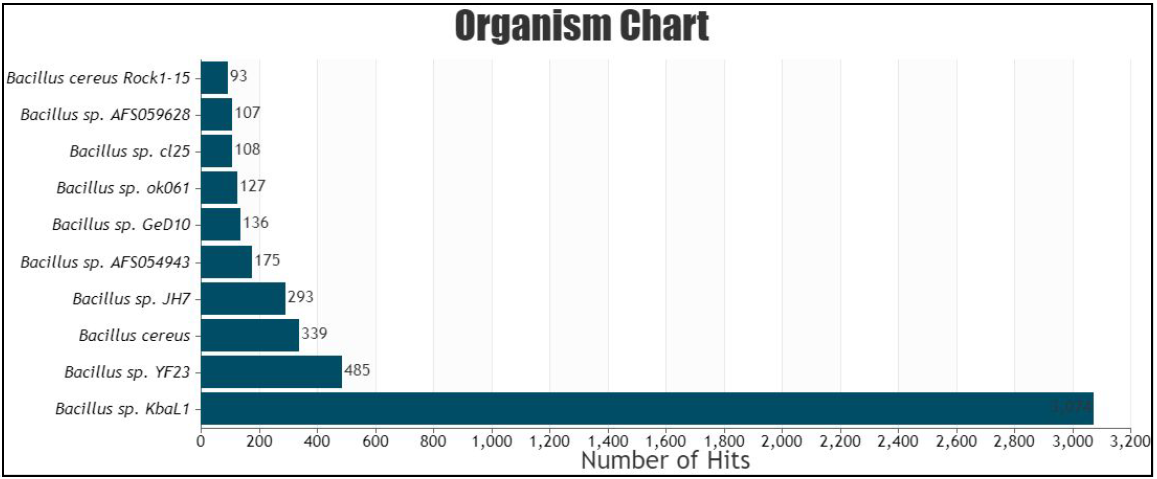


**Fig.S1.** BLASTX top 10 hit organism distribution of ‘BNPI-92’


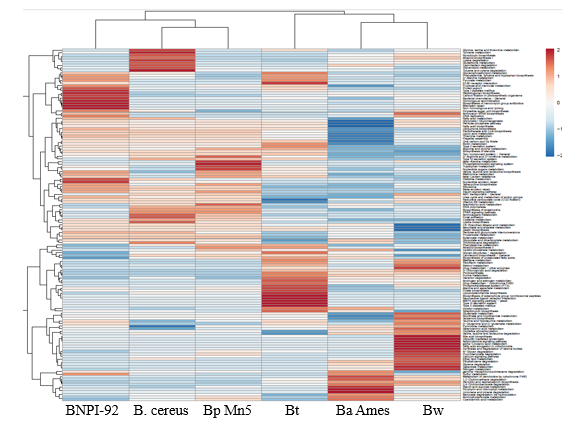


**Fig. S2**. Heatmap analysis for *Bacillus* sp. BNPI-92 and other related bacterial strains in terms of KEGG metabolic pathways or functional comparison with type of strains on X-axis and metabolic pathways on Y-axis. Z axis is a color pattern for distribution of gene encoded metabolic pathway that identified among these in Heatmap. "Z" is negative when the raw score is below the mean, positive when deep red indicate high level of protein expression. Heatmap was performed according to Metsalu, &Vilo, ^10^ and using online tools that available at <https://biit.cs.ut.ee/clustvis/>.

**Table S_1_** BLAST summery for BNPI-92 NGS.

| **No.** | **Sample Name** | **Predicted gene in BNPI-92 NGS** | |
| --- | --- | --- | --- |
| 1 | BNPI-92 | Number of predicted genes | 5,719 |
| 2 | “ | Number of predicted genes with significant  BLASTX match with UniProt | 5,652 |

**Table S_2_** OrthoVenn analysis summery for comparative genomic analysis

| **No** | **Strains** | **Proteins** | **Clusters** | **Singletons** |
| --- | --- | --- | --- | --- |
| 1 | *Bacillus* sp. BNPI-92 | 5657 | 5127 | 492 |
| 2 | *Bacillus cereus* ATCC 14579 | 5700 | 5118 | 515 |
| 3 | *Bacillus paranthracis* Mn5 | 5675 | 5045 | 577 |
| 4 | *Bacillus thuringiensis* ATCC 10792 | 5554 | 5122 | 350 |
| 5 | *Bacillus anthracis* Ames | 5642 | 5097 | 493 |
| 6 | *Bacillus wiedmannii* FSL w8-0169 | 4014 | 3657 | 318 |

Key note: The species form 5963 clusters, 3067 orthologous clusters (at least contains two species) and cluster contain only one protein for each species of single-copy gene clusters (2896). Singletons (indicates that proteins are not in any cluster).

**Table S_3_.**Gene Ontology terms identified in each category (UniPort/Ortho Venn)

| **No** | **Category** | **Number of terms** |
| --- | --- | --- |
| 1 | Biological Processes | 475/763 |
| 2 | Molecular Functions | 802/546 |
| 3 | Cellular Components | 69/264 |

**Table S_4_: Multilocus sequence typing (MLST)** results

| **Locus** | **Allele** | **Length** | **Contigs** | **Start position** | **End position** | ***Linked data values*** |
| --- | --- | --- | --- | --- | --- | --- |
| BACT000001 (*rpsA*) | 495 | 1149 | NODE_389_length_394067_cov_60.326180 | 84290 | 85438 | Species*: Bacillus cereus [n=98]; Bacillus sp. [n=17]; Bacillus thuringiensis [n=5]* |
| BACT000002 (*rpsB*) | 40 | 702 | NODE_7_length_698727_cov_62.297756 | 124503 | 125204 | Species*: Bacillus cereus [n=174]; Bacillus sp. [n=28]; Bacillus albus [n=10]; Bacillus thuringiensis [n=5]* |
| BACT000003 (*rpsC*) | 25 | 660 | NODE_66_length_58329_cov_95.510330 | 20428 | 21087 | Species*: Bacillus cereus [n=686]; Bacillus thuringiensis [n=154]; Bacillus sp. [n=59]; Bacillus pacificus [n=37]; Bacillus paranthracis [n=18]; Bacillus albus [n=9]; Bacillus bombysepticus [n=1]* |
| BACT000004 (*rpsD*) | 815 | 603 | NODE_121_length_194373_cov_67.235977 | 30651 | 31253 | Species*: Bacillus cereus [n=20]; Bacillus sp. [n=6]; Bacillus thuringiensis [n=4]* |
| BACT000005 (*rpsE*) | 24 | 501 | NODE_66_length_58329_cov_95.510330 | 16055 | 16555 | *species: Bacillus cereus [n=595]; Bacillus thuringiensis [n=144]; Bacillus sp. [n=46]; Bacillus bombysepticus [n=1]* |
| BACT000006 (*rpsF*) | 29 | 291 | NODE_238_length_183151_cov_79.094261 | 21883 | 22173 | Species*: Bacillus cereus [n=456]; Bacillus thuringiensis [n=135]; Bacillus sp. [n=39]; Bacillus pacificus [n=34]; Bacillus albus [n=9]; Bacillus tropicus [n=2]; Bacillus bombysepticus [n=1]; Bacillus fungorum [n=1]; Bacillus paranthracis [n=1]* |
| BACT000007 (*rpsG*) | 23 | 471 | NODE_66_length_58329_cov_95.510330 | 28553 | 29023 | Species*: Bacillus cereus [n=556]; Bacillus thuringiensis [n=89]; Bacillus sp. [n=40]; Bacillus bombysepticus [n=1]* |
| BACT000008 (*rpsH*) | 17 | 399 | NODE_66_length_58329_cov_95.510330 | 17543 | 17941 | Species*: Bacillus cereus [n=734]; Bacillus thuringiensis [n=527]; Bacillus anthracis [n=353]; Bacillus sp. [n=113]; Bacillus tropicus [n=9]; Bacillus mycoides [n=4]; Bacillus bombysepticus [n=1]* |
| BACT000009 (*rpsI*) | 27 | 393 | NODE_66_length_58329_cov_95.510330 | 5524 | 5916 | Species*: Bacillus cereus [n=888]Bacillus thuringiensis [n=520]Bacillus wiedmannii [n=190]Bacillus sp. [n=155]Bacillus pacificus [n=37]Bacillus paranthracis [n=32]Bacillus mobilis [n=15]Bacillus mycoides [n=13]Bacillus albus [n=10]Bacillus tropicus [n=9]Bacillus anthracis [n=4]Bacillus luti [n=4]Bacillus paramycoides [n=3]Bacillus paramobilis [n=2]Bacillus bombysepticus [n=1]Bacillus fungorum [n=1]Bacillus hominis [n=1]Bacillus sanguinis [n=1]* |
| BACT000010 (*rpsJ*) | 30 | 309 | NODE_66_length_58329_cov_95.510330 | 24254 | 24562 | Species*: Bacillus cereus [n=635]; Bacillus thuringiensis [n=147]; Bacillus sp. [n=60]; Bacillus paranthracis [n=32]; Bacillus pacificus [n=26]; Bacillus tropicus [n=9]; Bacillus bombysepticus [n=1]* |
| BACT000011 (*rpsK*) | 19 | 390 | NODE_66_length_58329_cov_95.510330 | 11394 | 11783 | Species*: Bacillus cereus [n=604]; Bacillus thuringiensis [n=148]; Bacillus sp. [n=49]; Bacillus luti [n=3]; Bacillus tropicus [n=2]; Bacillus bombysepticus [n=1]; Bacillus fungorum [n=1]* |
| BACT000012 (*rpsL*) | 28 | 423 | NODE_66_length_58329_cov_95.510330 | 29053 | 29475 | Species*: Bacillus cereus [n=595]; Bacillus thuringiensis [n=145]; Bacillus sp. [n=44]; Bacillus bombysepticus [n=1]* |
| BACT000013 (*rpsM*) | 19 | 366 | NODE_66_length_58329_cov_95.510330 | 11808 | 12173 | Species*: Bacillus cereus [n=522]; Bacillus anthracis [n=353]; Bacillus thuringiensis [n=148]; Bacillus sp. [n=62]; Bacillus tropicus [n=3]; Bacillus fungorum [n=1]* |
| BACT000014 (*rpsN*) | 16 | 186 | NODE_66_length_58329_cov_95.510330 | 17971 | 18156 | *species: Bacillus cereus [n=804]Bacillus thuringiensis [n=539]Bacillus anthracis [n=353]Bacillus toyonensis [n=229]Bacillus sp. [n=145]Bacillus albus [n=10]Bacillus tropicus [n=9]Bacillus luti [n=4]Bacillus mobilis [n=3]Bacillus paramycoides [n=3]Bacillus clarus [n=2]Bacillus bombysepticus [n=1]Bacillus fungorum [n=1]Bacillus pseudomycoides [n=1]Bacillus sanguinis [n=1]* |
| BACT000015 (*rpsO*) | 21 | 270 | NODE_7_length_698727_cov_62.297756 | 104179 | 104448 | Species*: Bacillus cereus [n=237]; Bacillus toyonensis [n=232]; Bacillus sp. [n=38]; Bacillus thuringiensis [n=18]* |
| BACT000016 (*rpsP*) | 21 | 273 | NODE_7_length_698727_cov_62.297756 | 140682 | 140954 | *species: Bacillus cereus [n=609]; Bacillus thuringiensis [n=359]; Bacillus sp. [n=63]; Bacillus bombysepticus [n=1]* |
| BACT000017 (*rpsQ*) | 21 | 264 | NODE_66_length_58329_cov_95.510330 | 19518 | 19781 | Species*: Bacillus cereus [n=801]; Bacillus thuringiensis [n=540]; Bacillus anthracis [n=351]; Bacillus toyonensis [n=230]; Bacillus sp. [n=135]; Bacillus tropicus [n=9]; Bacillus luti [n=4]; Bacillus bombysepticus [n=1]* |
| BACT000018 (*rpsR*) | 352 | 234 | NODE_238_length_183151_cov_79.094261 | 22758 | 22991 | Species*: Bacillus cereus [n=27]; Bacillus sp. [n=5]; Bacillus thuringiensis [n=3]* |
| BACT000019 (*rpsS*) | 19 | 279 | NODE_66_length_58329_cov_95.510330 | 21450 | 21728 | Species*: Bacillus cereus [n=535]; Bacillus thuringiensis [n=148]; Bacillus sp. [n=44]; Bacillus bombysepticus [n=1]* |
| BACT000020 (*rpsT*) | 287 | 258 | NODE_7_length_698727_cov_62.297756 | 601435 | 601692 | Species*: Bacillus cereus [n=901]Bacillus anthracis [n=343]Bacillus toyonensis [n=232]Bacillus thuringiensis [n=173]Bacillus sp. [n=112]Bacillus pacificus [n=36]Bacillus paranthracis [n=32]Bacillus tropicus [n=8]Bacillus nitratireducens [n=7]Bacillus luti [n=4]Bacillus paramycoides [n=3]Bacillus clarus [n=2]Bacillus bombysepticus [n=1]Bacillus proteolyticus [n=1]Bacillus sanguinis [n=1]* |
| BACT000021 (*rpsU*) | 16 | 174 | NODE_7_length_698727_cov_62.297756 | 587332 | 587505 | Species*: Bacillus cereus [n=977]Bacillus thuringiensis [n=546]Bacillus anthracis [n=351]Bacillus toyonensis [n=232]Bacillus sp. [n=180]Bacillus wiedmannii [n=126]Bacillus pacificus [n=37]Bacillus paranthracis [n=32]Bacillus albus [n=10]Bacillus tropicus [n=9]Bacillus mobilis [n=7]Bacillus pseudomycoides [n=6]Bacillus luti [n=4]Bacillus paramycoides [n=3]Bacillus bingmayongensis [n=2]Bacillus clarus [n=2]Bacillus bombysepticus [n=1]Bacillus fungorum [n=1]Bacillus sanguinis [n=1]* |
| BACT000030 (*rplA*) | 448 | 693 | NODE_66_length_58329_cov_95.510330 | 39263 | 39955 | Species*: Bacillus cereus [n=61]; Bacillus sp. [n=13]; Bacillus thuringiensis [n=1]* |
| BACT000031 (*rplB*) | 35 | 831 | NODE_66_length_58329_cov_95.510330 | 21789 | 22619 | Species*: Bacillus cereus [n=133]; Bacillus sp. [n=20]; Bacillus thuringiensis [n=3]* |
| BACT000032 (*rplC*) | 45 | 633 | NODE_66_length_58329_cov_95.510330 | 23587 | 24219 | Species*: Bacillus cereus [n=467]; Bacillus sp. [n=41]; Bacillus thuringiensis [n=30]; Bacillus bombysepticus [n=1]* |
| BACT000033 (*rplD*) | 32 | 624 | NODE_66_length_58329_cov_95.510330 | 22938 | 23561 | *species: Bacillus anthracis [n=352]; Bacillus cereus [n=217]; Bacillus thuringiensis [n=200]; Bacillus sp. [n=57]; Bacillus albus [n=8]; Bacillus luti [n=3]; Bacillus sanguinis [n=1]* |
| BACT000034 (*rplE*) | 31 | 540 | NODE_66_length_58329_cov_95.510330 | 18190 | 18729 | Species*: Bacillus cereus [n=534]; Bacillus thuringiensis [n=93]; Bacillus sp. [n=39]; Bacillus bombysepticus [n=1]; Bacillus tropicus [n=1]* |
| BACT000035 (*rplF*) | 30 | 540 | NODE_66_length_58329_cov_95.510330 | 16971 | 17510 | Species*: Bacillus cereus* [n=549]; *Bacillus thuringiensis* [n=93]; *Bacillus sp.* [n=47] |
| BACT000036 (*rplL*) | 28 | 360 | NODE_66_length_58329_cov_95.510330 | 38102 | 38461 | *species: Bacillus cereus [n=841]Bacillus anthracis [n=337]Bacillus thuringiensis [n=316]Bacillus sp. [n=118]Bacillus pacificus [n=37]Bacillus paranthracis [n=30]Bacillus tropicus [n=9]Bacillus albus [n=5]Bacillus bombysepticus [n=1]Bacillus pseudomycoides [n=1]Bacillus sanguinis [n=1]* |
| BACT000038 (*rplI*) | 367 | 447 | NODE_238_length_183151_cov_79.094261 | 26055 | 26501 | *species: Bacillus cereus [n=63]; Bacillus sp. [n=10]; Bacillus thuringiensis [n=6]* |
| BACT000039 (*rplJ*) | 341 | 501 | NODE_66_length_58329_cov_95.510330 | 38529 | 39029 | *species: Bacillus cereus [n=457]; Bacillus thuringiensis [n=148]; Bacillus sp. [n=36]; Bacillus bombysepticus [n=1]* |
| BACT000040 (*rplK*) | 36 | 426 | NODE_66_length_58329_cov_95.510330 | 40131 | 40556 | Species*: Bacillus cereus [n=867]; Bacillus thuringiensis [n=364]; Bacillus toyonensis [n=229]; Bacillus sp. [n=97]; Bacillus pacificus [n=36]; Bacillus paranthracis [n=28]; Bacillus tropicus [n=9]; Bacillus nitratireducens [n=7]; Bacillus bombysepticus [n=1]* |
| BACT000042 (*rplM*) | 27 | 438 | NODE_66_length_58329_cov_95.510330 | 5938 | 6375 | Species*: Bacillus cereus [n=601]; Bacillus thuringiensis [n=149]; Bacillus sp. [n=48]; Bacillus bombysepticus [n=1]* |
| BACT000043 (*rplN*) | 12 | 369 | NODE_66_length_58329_cov_95.510330 | 19106 | 19474 | Species*: Bacillus thuringiensis [n=379]; Bacillus cereus [n=315]; Bacillus sp. [n=92]; Bacillus tropicus [n=9]; Bacillus anthracis [n=3]; Bacillus toyonensis [n=3]; Bacillus fungorum [n=1]* |
| BACT000044 (*rplO*) | 24 | 441 | NODE_66_length_58329_cov_95.510330 | 15385 | 15825 | *species: Bacillus cereus [n=579]; Bacillus thuringiensis [n=93]; Bacillus sp. [n=46]; Bacillus bombysepticus [n=1]* |
| BACT000045 (*rplP*) | 19 | 435 | NODE_66_length_58329_cov_95.510330 | 19992 | 20426 | Species*: Bacillus cereus [n=660]; Bacillus thuringiensis [n=195]; Bacillus sp. [n=69]; Bacillus albus [n=9]; Bacillus bombysepticus [n=1]; Bacillus fungorum [n=1]; Bacillus paranthracis [n=1]* |
| BACT000046 (*rplQ*) | 25 | 363 | NODE_66_length_58329_cov_95.510330 | 9871 | 10233 | Species*: Bacillus cereus [n=593]; Bacillus thuringiensis [n=142]; Bacillus sp. [n=47]; Bacillus bombysepticus [n=1]; Bacillus fungorum [n=1]* |
| BACT000047 (*rplR*) | 19 | 363 | NODE_66_length_58329_cov_95.510330 | 16577 | 16939 | Species*: Bacillus cereus [n=665]; Bacillus anthracis [n=348]; Bacillus thuringiensis [n=172]; Bacillus sp. [n=85]; Bacillus albus [n=10]; Bacillus tropicus [n=9]; Bacillus bombysepticus [n=1]* |
| BACT000048 (*rplS*) | 29 | 345 | NODE_7_length_698727_cov_62.297756 | 138578 | 138922 | Species*: Bacillus cereus [n=599]; Bacillus thuringiensis [n=152]; Bacillus sp. [n=49]; Bacillus bombysepticus [n=1]* |
| BACT000049 (*rplT*) | 9187 | 357 | NODE_121_length_194373_cov_67.235977 | 113300 | 113656 | *species: Bacillus cereus [n=17]; Bacillus thuringiensis [n=14]; Bacillus sp. [n=6]; Bacillus luti [n=3]; Bacillus anthracis [n=2]* |
| BACT000050 (*rplU*) | 24 | 309 | NODE_29_length_75394_cov_65.720772 | 57806 | 58114 | Species*: Bacillus cereus [n=247]; Bacillus thuringiensis [n=215]; Bacillus wiedmannii [n=148]; Bacillus sp. [n=70]; Bacillus mobilis [n=15]; Bacillus albus [n=10]; Bacillus luti [n=4]; Bacillus paramycoides [n=3]; Bacillus fungorum [n=1]* |
| BACT000051 (*rplV*) | 21 | 342 | NODE_66_length_58329_cov_95.510330 | 21091 | 21432 | Species*: Bacillus cereus [n=244]; Bacillus sp. [n=26]; Bacillus thuringiensis [n=18]* |
| BACT000052 (rplW) | 34 | 291 | NODE_66_length_58329_cov_95.510330 | 22648 | 22938 | Species*: Bacillus cereus [n=752]Bacillus thuringiensis [n=480]Bacillus anthracis [n=350]Bacillus toyonensis [n=230]Bacillus sp. [n=144]Bacillus wiedmannii [n=21]Bacillus mobilis [n=15]Bacillus luti [n=4]Bacillus albus [n=2]Bacillus paramobilis [n=2]Bacillus bombysepticus [n=1]Bacillus fungorum [n=1]Bacillus paranthracis [n=1]Bacillus sanguinis [n=1]* |
| BACT000053 (rplX) | 26 | 312 | NODE_66_length_58329_cov_95.510330 | 18756 | 19067 | Species*: Bacillus cereus [n=223]; Bacillus sp. [n=34]; Bacillus thuringiensis [n=14]; Bacillus albus [n=10]; Bacillus tropicus [n=9]; Bacillus fungorum [n=1]; Bacillus sanguinis [n=1]* |
| BACT000056 (*rpmA*) | 35 | 291 | NODE_29_length_75394_cov_65.720772 | 58474 | 58764 | Species*: Bacillus cereus [n=541]; Bacillus thuringiensis [n=141]; Bacillus sp. [n=38]; Bacillus bombysepticus [n=1]* |
| BACT000057 (*rpmB*) | 19 | 189 | NODE_7_length_698727_cov_62.297756 | 156851 | 157039 | *species: Bacillus cereus [n=489]; Bacillus thuringiensis [n=479]; Bacillus sp. [n=114]; Bacillus pacificus [n=37]; Bacillus paranthracis [n=32]; Bacillus albus [n=10]; Bacillus tropicus [n=9]; Bacillus anthracis [n=4]; Bacillus sanguinis [n=1]* |
| BACT000058 (*rpmC*) | 10 | 201 | NODE_66_length_58329_cov_95.510330 | 19802 | 20002 | Species*: Bacillus cereus [n=800]Bacillus thuringiensis [n=528]Bacillus toyonensis [n=230]Bacillus sp. [n=131]Bacillus albus [n=10]Bacillus tropicus [n=9]Bacillus luti [n=4]Bacillus anthracis [n=3]Bacillus bombysepticus [n=1]Bacillus fungorum [n=1]Bacillus sanguinis [n=1]* |
| BACT000059 (*rpmD*) | 15 | 183 | NODE_66_length_58329_cov_95.510330 | 15859 | 16041 | Species*: Bacillus cereus [n=804]Bacillus anthracis [n=349]Bacillus thuringiensis [n=176]Bacillus pseudomycoides [n=111]Bacillus sp. [n=105]Bacillus pacificus [n=37]Bacillus paranthracis [n=32]Bacillus albus [n=9]Bacillus tropicus [n=9]Bacillus gaemokensis [n=2]Bacillus bombysepticus [n=1]* |
| BACT000060 (rpmE) | 19 | 246 | NODE_238_length_183151_cov_79.094261 | 181982 | 182227 | Species*: Bacillus cereus [n=668]; Bacillus anthracis [n=352]; Bacillus thuringiensis [n=166]; Bacillus sp. [n=87]; Bacillus albus [n=10]; Bacillus tropicus [n=6]; Bacillus bombysepticus [n=1]; Bacillus fungorum [n=1]; Bacillus sanguinis [n=1]* |
| BACT000061 (*rpmF*) | 19 | 174 | NODE_7_length_698727_cov_62.297756 | 226390 | 226563 | Species*: Bacillus cereus [n=1055]Bacillus thuringiensis [n=538]Bacillus anthracis [n=333]Bacillus toyonensis [n=232]Bacillus sp. [n=211]Bacillus wiedmannii [n=189]Bacillus mycoides [n=122]Bacillus pseudomycoides [n=111]Bacillus pacificus [n=37]Bacillus paranthracis [n=32]Bacillus mobilis [n=15]Bacillus albus [n=10]Bacillus tropicus [n=9]Bacillus nitratireducens [n=7]Bacillus luti [n=4]Bacillus paramycoides [n=3]Bacillus bingmayongensis [n=2]Bacillus clarus [n=2]Bacillus gaemokensis [n=2]Bacillus paramobilis [n=2]Bacillus bombysepticus [n=1]Bacillus fungorum [n=1]Bacillus hominis [n=1]Bacillus proteolyticus [n=1]Bacillus sanguinis [n=1]* |
| BACT000062 (*rpmG*) | 400 | 150 | NODE_7_length_698727_cov_62.297756 | 546522 | 546671 | Species*: Bacillus cereus [n=794]; Bacillus thuringiensis [n=515]; Bacillus toyonensis [n=229]; Bacillus sp. [n=109]; Bacillus mycoides [n=82]; Bacillus nitratireducens [n=7]; Bacillus bombysepticus [n=1]; Bacillus hominis [n=1]; Bacillus paramycoides [n=1]* |
| BACT000062 (*rpmG*) | 372 | 150 | NODE_7_length_698727_cov_62.297756 | 618158 | 618307 | Species*: Bacillus cereus [n=103]; Bacillus sp. [n=14]; Bacillus thuringiensis [n=6]* |
| BACT000062 (*rpmG*) | 7034 | 147 | NODE_66_length_58329_cov_95.510330 | 41601 | 41747 | Species*: Bacillus cereus [n=629]; Bacillus thuringiensis [n=441]; Bacillus sp. [n=78]; Bacillus bombysepticus [n=1]* |
| BACT000063 (*rpmH*) | 21 | 135 | NODE_238_length_183151_cov_79.094261 | 9444 | 9578 | Species*: Bacillus cereus [n=1091]Bacillus thuringiensis [n=546]Bacillus toyonensis [n=232]Bacillus sp. [n=199]Bacillus wiedmannii [n=190]Bacillus pseudomycoides [n=112]Bacillus pacificus [n=34]Bacillus paranthracis [n=31]Bacillus cytotoxicus [n=21]Bacillus mobilis [n=15]Bacillus albus [n=10]Bacillus tropicus [n=9]Bacillus nitratireducens [n=7]Bacillus anthracis [n=4]Bacillus luti [n=4]Bacillus paramycoides [n=3]Bacillus bingmayongensis [n=2]Bacillus clarus [n=2]Bacillus gaemokensis [n=2]Bacillus paramobilis [n=2]Bacillus bombysepticus [n=1]Bacillus fungorum [n=1]Bacillus hominis [n=1]Bacillus proteolyticus [n=1]Bacillus sanguinis [n=1]* |
| BACT000064 (*rpmI*) | 14 | 201 | NODE_121_length_194373_cov_67.235977 | 113062 | 113262 | Species*: Bacillus cereus [n=1028]Bacillus thuringiensis [n=546]Bacillus anthracis [n=353]Bacillus toyonensis [n=232]Bacillus sp. [n=153]Bacillus pacificus [n=37]Bacillus paranthracis [n=32]Bacillus albus [n=10]Bacillus tropicus [n=9]Bacillus nitratireducens [n=7]Bacillus luti [n=3]Bacillus bombysepticus [n=1]Bacillus proteolyticus [n=1]Bacillus sanguinis [n=1]* |
| BACT000065 (*rpmJ*) | 11 | 114 | NODE_66_length_58329_cov_95.510330 | 12195 | 12308 | Species*: Bacillus cereus [n=1090]Bacillus thuringiensis [n=546]Bacillus anthracis [n=353]Bacillus toyonensis [n=232]Bacillus sp. [n=222]Bacillus wiedmannii [n=190]Bacillus mycoides [n=122]Bacillus pseudomycoides [n=112]Bacillus pacificus [n=37]Bacillus paranthracis [n=32]Bacillus cytotoxicus [n=21]Bacillus mobilis [n=15]Bacillus albus [n=10]Bacillus tropicus [n=9]Bacillus nitratireducens [n=7]Bacillus luti [n=4]Bacillus paramycoides [n=3]Bacillus bingmayongensis [n=2]Bacillus clarus [n=2]Bacillus gaemokensis [n=2]Bacillus paramobilis [n=2]Bacillus bombysepticus [n=1]Bacillus fungorum [n=1]Bacillus hominis [n=1]Bacillus proteolyticus [n=1]Bacillus sanguinis [n=1]* |
